# Robust Amphiphobic Few‐Layer Black Phosphorus Nanosheet with Improved Stability

**DOI:** 10.1002/advs.201901991

**Published:** 2019-09-30

**Authors:** Xiao Liu, Yunfei Bai, Jun Xu, Qingchi Xu, Liangping Xiao, Liping Sun, Jian Weng, Yanli Zhao

**Affiliations:** ^1^ Department of Biomaterials College of Materials Xiamen University Xiamen 361005 P. R. China; ^2^ Department of Physics Research Institute for Biomimetics and Soft Matter Fujian Provincial Key Laboratory for Soft Functional Materials Xiamen University Xiamen 361005 P. R. China; ^3^ Shenzhen Research Institute of Xiamen University Shenzhen 518057 P. R. China; ^4^ Division of Chemistry and Biological Chemistry School of Physical and Mathematical Sciences Nanyang Technological University 21 Nanyang Link 637371 Singapore Singapore; ^5^ State Key Lab of Physical Chemistry of Solid Surfaces Collaborative Innovation Center of Chemistry for Energy Materials College of Chemistry and Chemical Engineering Xiamen University Xiamen 361005 P. R. China

**Keywords:** amphiphobic surfaces, black phosphorus, enhanced stability, fluorinated coating, nanosheets

## Abstract

Few‐layer black phosphorus (FL‐BP) has been intensively studied due to its attractive properties and great potential in electronic and optoelectronic applications. However, the intrinsic instability of FL‐BP greatly limits its practical application. In this study, the amphiphobic FL‐BP is achieved by functionalization of 1*H*,1*H*,2*H*,2*H*‐perfluorooctyltrichlorosilane (PFDTS) on the surface of FL‐BP. The obtained PFDTS coated FL‐BP (FL‐BP/PFDTS) demonstrates enhanced stability, which is not observed during significant degradation for 2 months in high moisture content environment (95% humidity). Particularly, attributing to the surface amphiphobicity, FL‐BP/PFDTS exhibits strong surface water repellency in the presence of oleic acid (as the contaminant), while other passivation coating layers (such as hydrophilic or hydrophobic coating) become hydrophilicity under such conditions. Owing to this advantage, the obtained FL‐BP/PFDTS demonstrates enhanced stability in high moisture content environment for 2 months, even though the surface is contaminated by oil liquid or other organic solvents (such as oleic acid, CH_2_Cl_2_, and *N*‐methyl‐2‐pyrrolidone). The passivation of FL‐BP by amphiphobic coating provides an effective approach for FL‐BP stabilization toward future applications.

## Graphical abstract

Amphiphobic few‐layer black phosphorus is developed by the surface functionalization of 1*H*,1*H*,2*H*,2*H*‐perfluorooctyltrichlorosilane, exhibiting significantly enhanced stability in high moisture content environment for 2 months, even though the surface is contaminated by oils or organic solvents.

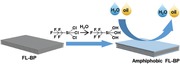

## 1 Introduction

Electronic and optoelectronic devices are absolutely indispensable part of information and communication technology in the 21st century. The few‐layer black phosphorus (FL‐BP), as a rising star among various 2D nanomaterials, has been intensively investigated to apply in electronic and optoelectronic device since early 2014.^1^ Distinct from graphene and other 2D materials, FL‐BP features highly anisotropic charge‐transport and optical‐response properties.^2^ Particularly, BP has a highly thickness‐dependent bandgap, which is 0.3 eV for bulk BP and 1.8–2.0 eV for monolayer respectively.^3^ Moreover, FL‐BP also demonstrates large on/off ratio (>10^5^) and high charger mobility (1000 cm^2^ V^−1^ s^−1^).^4^ FL‐BP is considered to be a promising material for electronic and optoelectronic devices and attracts widespread attention on account of such unique and attractive properties.^5^

Unfortunately, although black phosphorus is the most thermodynamically stable allotrope of phosphorus,^6^ FL‐BP is susceptible to degrade upon exposure to ambient environment as monolayer BP, and even FL‐BP may degrade within hours.^7^ The practical application of FL‐BP on electronic and optoelectronic devices is greatly limited due to its instability under ambient conditions.

While the detailed mechanism of FL‐BP degradation in air remains to be further revealed, the experimental and theoretical evidence available up to now shows that the synergetic effect of water and oxygen plays an important role.^8^ The oxygen chemosorbs on the surface of FL‐BP, subsequently reacts with P to form P*_x_*O*_y_*. Then, water molecules attach with P*_x_*O*_y_* through hydrogen bond and destroy the P network of FL‐BP.[qv: 8c] It is supposed to be an effective strategy to stabilize FL‐BP under ambient conditions if researchers can prevent oxygen/water attachment on BP surface.

Under the guidance of this idea, several types of materials are employed as coating layer for the passivation of FL‐BP, such as hydrophilic layers (e.g., titanium sulfonate, ionic liquid, lactic‐*co*‐glycolic acid, and polyethylene glycol),^9^ hydrophobic layers (e.g., aryl diazonium, electrolyzed fluorine ions, 7,7,8,8‐tetracyano‐*p*‐quinodimethane, polymeric stabilizer, and azodiisobutyronitrile),^10^ as well as solvation shells (1‐methyl‐2‐pyrrolidone and *N*‐cyclohexyl‐2‐pyrrolidone).^11^ The coated FL‐BP can be stabilized at ambient conditions for 100 days (hydrophilic layer)[qv: 9e] and even longer than 200 days (hydrophobic layer).[qv: 10f] Usually, hydrophobic coating renders FL‐BP with higher stability than that of hydrophilic coating due to its surface water repellency. However, such hydrophobic coating loses its water repellency when it is contaminated by oil liquids or other organic solvents. It is conceivable that the hydrophobically functionalized FL‐BP becomes less stable once it undergoes such oil contamination and loses its water repellency.^12^ Actually, it seems that FL‐BP cannot totally avoid such contamination in electronics and optical practice. Therefore, although thin layer coating is definitely an effective approach for FL‐BP stability enhancement, more effective passivation methodologies for FL‐BP stabilization are still in demand.^13^

Creating robust amphiphobic coating to repel both oil and water is one of the best strategies to solve such problem. There has been significant progress in making amphiphobic surface on various substrate (such as TiO_2_ and SiO_2_), and the obtained coating layer demonstrates highly water and oil repellent property in the presence of oil or organic solvent contaminants.[qv: 12b,14] Most importantly, the amphiphobic layer can effectively prevent the attachment of oil liquids or other organic solvents on its surface. Therefore, the contamination is restricted, and the surface water repellency preserves. The capability of preserving water repellency by amphiphobic coating contributes to the enhanced stability of the modified substrates.^15^ However, such amphiphobic functionalization has barely been extended to FL‐BP. It is highly desired to functionalize FL‐BP with robust amphiphobicity to enhance the stability through the rendered high water repellency even under various contaminating conditions.

**Scheme 1:**
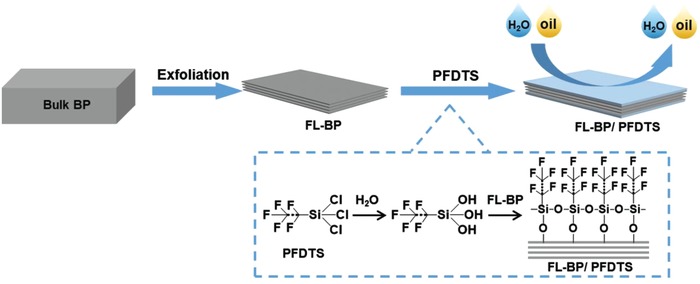
Schematic illustration of the exfoliation of bulk BP to FL‐BP and the subsequent functionalization of PFDTS on the FL‐BP surface.

In this paper, we report robust amphiphobic FL‐BP, demonstrating enhanced stability in high moisture content environment (95% humidity), even its surface being contaminated by oil liquids or organic solvents (**Scheme** 1). Fluorinated coating is commonly utilized for giving hydrophobic/oleophobic (amphiphobic) surface.^16^ Although the key factors for achieving amphiphobicity by fluorinated coating are not clear yet, the low surface energy arising from high concentration of ‐CF_2_ and ‐CF_3_ groups on its surface is essential for such purpose.[qv: 12a,d] In our system, 1*H*,1*H*,2*H*,2*H*‐perfluorooctyltrichlorosilane (PFDTS) is ideally utilized as fluorinated coatings, as it has abundant ‐CF_2_ and ‐CF_3_ groups on its backbone. First, PFDTS hydrolyze and form silanol‐containing species. Then, the uniform amphiphobic layer on the surface of FL‐BP is covalently grafted by reaction between the hydroxyl groups and P*_x_*O*_y_*, (from the slight oxidization during exfoliation).^17^ The amphiphobic coating significantly contributes to the stability of the obtained PFDTS functionalized FL‐BP (FL‐BP/PFDTS) in high moisture content environment. More importantly, amphiphobic coating imposes the capability to preserve the water repellency when various contaminants (such as oleic acid, CH_2_Cl_2_, and *N*‐methyl‐2‐pyrrolidone) are introduced. The obtained FL‐BP/PFDTS showed no significant degradation under high moisture content environment for 2 months, even though its surface was contaminated.

## 2 Results and Discussion

Bulk BP are prepared from red phosphorus via a facile low‐pressure transport route (the same method as we previously reported),^18^ and further characterized (Figure S1, Supporting Information). Scanning electron microscope (SEM) image of the as‐prepared bulk BP indicates the layered structure (Figure S1b, Supporting Information). Powder X‐ray diffraction (XRD) characterization shows the diffraction peaks of bulk BP (Figure S1c, Supporting Information), which can be indexed to the orthorhombic structure of BP (JCPDS card no. 73‐1385). The diffractions peaking at 17.34°, 34.66°, and 52.72° are appropriately indexed to the bulk BP at the positions of (020), (040), and (060) planes. Such results demonstrate the successful preparation of highly crystalline bulk BP.

The FL‐BP was obtained by sonication (24 h) associated exfoliation of bulk BP in *N*,*N*‐dimethylformamide (DMF). By tuning the speed of centrifugation (4000, 8000, and 12 000 rpm), three batches of exfoliated FL‐BP with 1057 ± 105 nm, 566 ± 72 nm, and 222 ± 33 nm in size, and 10.39 ± 0.58 nm, 2.64 ± 0.26 nm, and 1.24 ± 0.24 nm in thickness are obtained based on TEM and AFM observation (Figure S2a,b, Supporting Information), respectively. UV–vis spectra and polarizing microscope were used for addressing the degradation of these three batches of FL‐BP. FL‐BP nanosheets with size of 1057 ± 105 nm and thickness of 10.39 ± 0.58 nm showed significant degradation in aqueous solution by UV characterization after 9 days (more details in Figure S2 in the Supporting Information). FL‐BP nanosheets with size of 566 ± 72 nm and thickness of 2.64 ± 0.26 nm significantly degraded in aqueous solution after 7 days, while FL‐BP nanosheets with size of 222 ± 33 nm and thickness of 1.24 ± 0.24 nm exhibited the worst stability, showing significant degradation after 5 days (Figure S2d, Supporting Information). Obviously, as expected, the larger and thicker nanosheet is, the more ambient stability of FL‐BP nanosheet exhibits. For the largest and thickest FL‐BP nanosheets, they are relatively stable. Thus, they are unsuitable for making comparison with FL‐BP/PFDTS to show the improvement on the stability of nanosheets. On the other hand, for the smallest FL‐BP nanosheets, it is not easy to characterize the nanosheets due to the bad processability arising from its small size and poor stability. In terms of this issue, FL‐BP with 566 ± 72 nm in size (Figure S3a, Supporting Information) and 2.64 ± 0.26 nm in thickness (Figure S4a, Supporting Information) was chosen for the stability investigation because of the thin flakes and relatively large lateral area.^19^ Moreover, for most of the FL‐BP based applications, BP nanosheets with thickness around 10 nm, instead of monolayered BP, are of fundamental interest.[qv: 1a,11b,20]

Transmission electron microscope (TEM) images of typical FL‐BP show 2D nanostructure, with sizes of 566 ± 72 nm (Figures S3a and S5a, Supporting Information). The selected area electronic diffraction (SAED) shows single crystal structure of FL‐BP (inset of Figure S5a, Supporting Information). High‐resolution TEM (HRTEM) image reveals the lattice spacing of 2.6 and 3.5 Å, which can be assigned to the (040) and (021) plane of BP (Figure S5b, Supporting Information). The elemental distribution mapping of FL‐BP shows the uniform distribution of P and O over the whole nanosheet, indicating the slight oxidation on the surface of FL‐BP (Figure S5d, Supporting Information). AFM characterization reveals that the thickness of FL‐BP is 2.64 ± 0.26 nm (Figures S4a and S5c, Supporting Information), which is about 4–6 individual phosphorene layers.^21^

**Figure 1:**
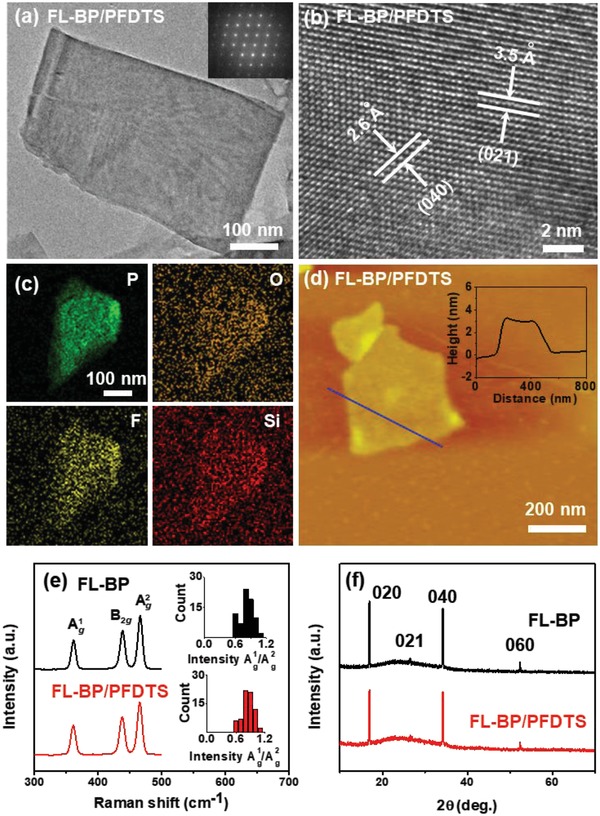
a) TEM (Inset: SAED pattern of FL‐BP/PFDTS), b) HRTEM, c) HAADF‐STEM and elemental mapping, and d) AFM (Inset: height profile along the black line) image of FL‐BP/PFDTS. e) Raman spectra (inset: histogram for the intensity ratio of $A_{g}^{1}/A_{g}^{2}$ mode) and f) powder XRD of the FL‐BP and the FL‐BP/PFDTS.

The FL‐BP/PFDTS is prepared by dispersing FL‐BP in PFDTS DMF solution for 24 h under vigorous stirring at room temperature. As PFDTS is easily hydrolyzed, it reacts with P*_x_*O*_y_* on the surface of FL‐BP, giving amphiphobic layer. **Figure** 1a is the TEM image of as‐prepared FL‐BP/PFDTS, showing a typical nanosheet structure and the size of 574 ± 48 nm by statistical TEM (Figure S3b, Supporting Information). HRTEM image (Figure 1b) discloses that the lattice fringe of FL‐BP/PFDTS is similar to the as‐prepared FL‐BP. Statistical AFM images show that the average thickness of FL‐BP/PFDTS is 3.71 ± 0.27 nm (Figure 1d; Figure S4b, Supporting Information). Such observation indicates that the morphology and SAED of FL‐BP/PFDTS is similar to the as‐prepared FL‐BP (Figure S5, Supporting Information), while increasing in the FL‐BP thickness provides evidence of covalent functionalization.[qv: 10a,22] High‐angle annular dark field‐scanning transmission electron microscopy (HAADF‐STEM) image of FL‐BP/PFDTS indicates that P, F, O, and Si elements are uniformly distributed on the whole FL‐BP/PFDTS nanosheet (Figure 1c), which further confirms the success of PFDTS functionalization on FL‐BP surface. The zeta potentials of FL‐BP and FL‐BP/PFDTS are −27.8 and −5.3 mV respectively (Figure S6, Supporting Information), also demonstrating the existence of PFDTS on the FL‐BP surface.

The structure of FL‐BP and FL‐BP/PFDTS is then investigated by Raman spectroscopy and XRD. As shown in Figure 1e, three Raman peaks at 362.5, 439.3, and 467.6 cm^−1^ are observed from the FL‐BP/PFDTS, which can be assigned to the $A_{g}^{1}$, *B*_2_ *_g_*, and $A_{g}^{2}$ modes respectively. Similarly, such peaks are also observed in Raman spectrum of FL‐BP.^23^ In addition, we analyzed the ratio of $A_{g}^{1}/A_{g}^{2}$ intensity by measuring 70 individual Raman spectra of FL‐BP and FL‐BP/PFDTS, respectively (inset of Figure 1e). The spectra of most samples show $A_{g}^{1}/A_{g}^{2}$ > 0.6, indicating the basal planes to be unoxidized completely.[qv: 5f,8a,11a] The Raman results demonstrate that FL‐BP/PFDTS maintain the structure of FL‐BP, which is further supported by the XRD. As shown in Figure 1f, XRD peaks attributed to FL‐BP remain in FL‐BP/PFDTS, indicating the crystalline BP structure survived after functionalization. Overall, despite the slight increase on its thickness, the functionalization with PFDTS has no significant effect on the 2D feature and properties of BP nanosheets.

**Figure 2:**
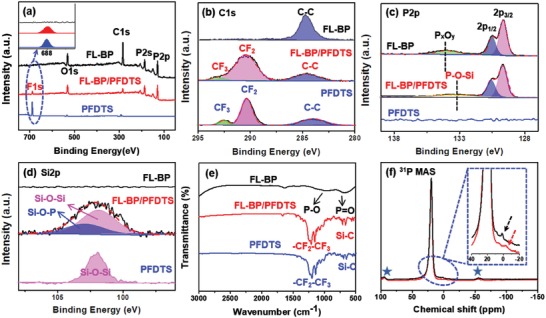
a) Full XPS spectra of FL‐BP, FL‐BP/PFDTS, and PFDTS (Inset: HR‐XPS spectra of F 1s peaks). HR‐XPS spectra of b) C 1s peaks, c) P 2p peaks, and d) Si 2p peaks. e) FTIR spectra of FL‐BP, FL‐BP/PFDTS, and PFDTS. f) ^31^P solid‐state NMR spectra of FL‐BP with black line and FL‐BP/PFDTS with red line (★ denotes the peaks of spinning sideband).

The X‐ray photoelectron spectroscopy (XPS) analysis is further carried out to analyze the surface compositions and chemical states of the elements present in FL‐BP/PFDTS (**Figure** 2). The binding energies in XPS spectra were calibrated using C 1s peak (284.6 eV). The complete spectra of FL‐BP and FL‐BP/PFDTS confirm the involvement of P, O, C and F, P, O, C elements, respectively (Figure 2a). It is noted that the peak at 284.6 eV can be attributed to the signal of adventitious carbon for FL‐BP and FL‐BP/PFDTS. The peaks corresponding to the CF_2_ and CF_3_ in high‐resolution XPS (HR‐XPS) spectra of C 1s for FL‐BP/PFDTS are observed at 290.5 and 293.1 eV, respectively (Figure 2b). These two peaks indicate the existence of PFDTS on the FL‐BP surface. In addition, HR‐XPS peak of F 1s at 687.4 eV further confirms successful functionalization of PFDTS on the FL‐BP surface (inset of Figure 2a).

The high‐resolution P 2p electron core‐level XPS spectra of FL‐BP and FL‐BP/PFDTS are further investigated in Figure 2c. The two main peaks observed from FL‐BP and FL‐BP/PFDTS at the binding energy position of 129.6 and 130.5 eV correspond to the P 2p_3/2_ and P 2p_1/2_, respectively, confirming the existence of P.^24^ The peak at 134.1 eV from FL‐BP is assigned to P*_x_*O*_y_*,^24^ whereas the peak with distinct lower signal at 133.4 eV from FL‐BP/PFDTS is assigned to P—O—Si.^25^ These results strongly suggest that PFDTS is functionalized on FL‐BP surface via P—O—Si bond. HR‐XPS of Si 2p provides further information about the formation of P–O–Si. The asymmetric peak of Si 2p can be divided into two peaks for FL‐BP/PFDTS: one is the peak of Si—O—P at 102.9 eV and the other is that of Si—O—Si at 102.1 eV (Figure 2d).^25^, ^26^ Based on XPS spectra, the atomic ratio of P/F in the FL‐BP/PFDTS is calculated as 1.1:1, which is in agreement with the theoretical calculation (1.36). In addition, the intensity of P—O—Si bond increases (from 4.0% to 47.2%) upon increasing the PFDTS concentration (from 4.0% to 47.2%), indicating more PFDTS functionalization on the BP surface (Figure S7 and Table S1, Supporting Information).

Fourier transform infrared spectroscopy (FTIR) analysis is conducted to further verify the formation of P—O—Si bond during the PFDTS functionalization. The FTIR of FL‐BP shows one weak characteristic band at the position of ≈1631.7 cm^−1^ (Figure 2e), assigned to H–O–H bending of water. The other two weak characteristic bands of FL‐BP are observed at the position of ≈979.2 and ≈669.9 cm^−1^, which can be ascribed to the P—O stretching vibration and the P=O bending vibration respectively.^23^, ^27^ Hence, the surface of FL‐BP has been oxidized partially during exfoliation. In FTIR spectrum of FL‐BP/PFDTS (Figure 2e), the peaks observed at ≈1246.3 and ≈1208.2 cm^−1^ are assigned to asymmetric and symmetric stretching of the ‐CF_2_‐ moiety, and the sharp band at 1157.4 cm^−1^ corresponds to the ‐CF_2_‐CF_3_ end group.^28^ The characteristic band around 658.2 and 712.4 cm^−1^ arises from the Si—C bonds.^29^ These bands are close to those of PFDTS (Figure 2e), indicating the presence of the long‐chain fluoroalkyl units on the FL‐BP surface. Furthermore, the peaks of 1200–1000 cm^−1^ of FL‐BP/PFDTS correspond to the Si—O—Si stretching vibration.^30^ The covalent bond of Si—O—Si is supposed to be evolved from hydrogen bonds between a pair of Si‐OH groups during PFDTS hydrolysis. The ^31^P solid‐state nuclear magnetic resonance (NMR) spectra of FL‐BP and FL‐BP/PFDTS were performed to further explore the chemical state of P atom. As shown in Figure 2f, a distinct peak at 19.8 ppm appears for both FL‐BP and FL‐BP/PFDTS, which is ascribed to the P—P bond.^31^ For FL‐BP/PFDTS, in addition to the signal for P—P bond, the new shoulder peak at −4.1 ppm is identified, which can be assigned to the Si—O—P bond.[qv: 31a,32] For FL‐BP, the shoulder peak at 1.1 ppm is also identified (inset of Figure 2f), which is probably assigned to the phosphorous acid. These results indicate the functionalization of FL‐BP/PFDTS via Si—O—P bond. The low intensity of the peak at −4.1 ppm indicates that probable noncovalent functionalization of PFDTS on the FL‐BP surface may exist beyond the covalent functionalization.

**Figure 3:**
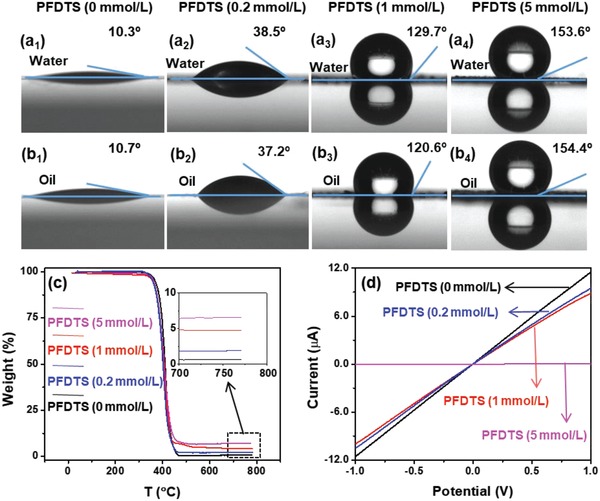
a) Water contact angles, b) oil contact angles, c) TG curves, and d) *I*/*V* curves of FL‐BP/PFDTS synthesized in the presence of different amount of PFDTS.

The fluorinated coating provides effective approach for achieving amphiphobic surface. Usually, the amphiphobic properties of the functionalized surface are greatly affected by the amount of fluorinated surfactant.^33^ Therefore, the effects of PFDTS concentration on the contact angle of FL‐BP/PFDTS are investigated (**Figure** 3). The water contact angle and oil contact angle of the initial FL‐BP (PFDTS 0 mol L^−1^) are 10.3° and 10.7° respectively (Figure 3a_1_,b_1_). As the concentration of PFDTS increases, the water and oil contact angle of FL‐BP/PFDTS turn into 38.5° and 37.2° (Figure 3a_2_,b_2_, PFDTS 0.2 mmol L^−1^), 129.7° and 120.6° (Figure 3a_3_,b_3_, PFDTS 1 mmol L^−1^), and 153.6° and 154.4° (Figure 3a_4_,b_4_, PFDTS 5 mmol L^−1^), respectively. Obviously, low concentration of PFDTS (<0.2 mmol L^−1^) does not render BP amphiphobicity, probably because of the partial coating of PFDTS on BP surface (Figures S8 and S9 for details in the Supporting Information). High concentration of PFDTS offers amphiphobic property (>90° for both water and oil contact angle) of FL‐BP/PFDTS (>1 mmol L^−1^), and even ultra‐amphiphobicity (>5 mmol L^−1^).

Thermogravimetric (TG) analysis reveals that FL‐BP/PFDTS (PFDTS 0 mmol L^−1^), FL‐BP/PFDTS (PFDTS 0.2 mmol L^−1^), FL‐BP/PFDTS (PFDTS 1 mmol L^−1^) and FL‐BP/PFDTS (PFDTS 5 mmol L^−1^) contain 0% SiO_2_, ≈1.5% SiO_2_, ≈4.9% SiO_2_ and ≈6.5% SiO_2_, respectively (Figure 3c). According to theoretical calculation (Figure S9, Supporting Information), PFDTS concentration of 0.2 mmol L^−1^ gives partially coated FL‐BP/PFDTS (<< 4.8% SiO_2_). PFDTS concentration of 1 mmol L^−1^ achieves thin layer of PFDTS on FL‐BP surface with full encapsulation (≈4.8% SiO_2_ and thickness of PFDTS is ≈2.6 Å). PFDTS concentration of 5 mmol L^−1^ results in thick layer coated FL‐BP (≈6.9% SiO_2_ and thickness of PFDTS is calculated as ≈13.0 Å). The TEM image of FL‐BP/PFDTS (PFDTS 5 mmol L^−1^) reveals the thick coating layer on FL‐BP surface (Figure S10, Supporting Information). The signal of P element, and diffraction peaks of FL‐BP cannot be detected by XPS or XRD, suggesting the thick PFDTS coating layer on the surface of FL‐BP.

Increasing concentration of PFDTS (>1 mol L^−1^) gives amphiphobic, even superamphiphobic surface of FL‐BP, but it also thickens the coating layer and decreases the conductivity of FL‐BP/PFDTS. Although PFDTS coating is insulating, the electron still can transfer through both bond and space,^34^ where the through‐space electron transfer greatly depends on the space‐distance.^35^ It is reported that through‐space electron transfer is dismissed when space‐distance is greater than 5 Å.^36^ The measured resistance for original FL‐BP is ≈0.8 × 10^5^ Ω (Figure 3d). The resistance of partially PFDTS coated FL‐BP (≈1 × 10^5^ Ω for PFDTS 0.2 mmol L^−1^) and thin layer fully encapsulated FL‐BP (≈1.2 × 10^5^ Ω for PFDTS 1 mmol L^−1^, thickness ≈ 2.6 Å) do not increase significantly. However, when comes to the case of fully thick layer encapsulated FL‐BP (PFDTS 5 mmol L^−1^, thickness ≈ 13.0 Å), the conductivity of FL‐BP/PFDTS (≈3.1 × 10^8^ Ω) decreases dramatically (Figure 3d).

The effect of surface properties on the stability of functionalized FL‐BP is further investigated. To make comparison, three types of functionalized FL‐BP, coated by 1) hydrophilic layer ((3‐aminopropyl)trimethoxysilane, FL‐BP/AMPTS), 2) hydrophobic layer (trichloro(dodecyl)silane, FL‐BP/DDTS), and 3) amphiphobic layer (FL‐BP/PFDTS), are prepared respectively. The structures of FL‐BP/AMPTS and FL‐BP/DDTS are characterized via FTIR and XPS, and the results confirm successful functionalization via Si—O—P bond (Figure S11, Supporting Information). By measuring the water and oil contact angle, FL‐BP/AMPTS presents hydrophilic property, and FL‐BP/DDTS shows hydrophobic property (Figure S12, Supporting Information).

**Figure 4:**
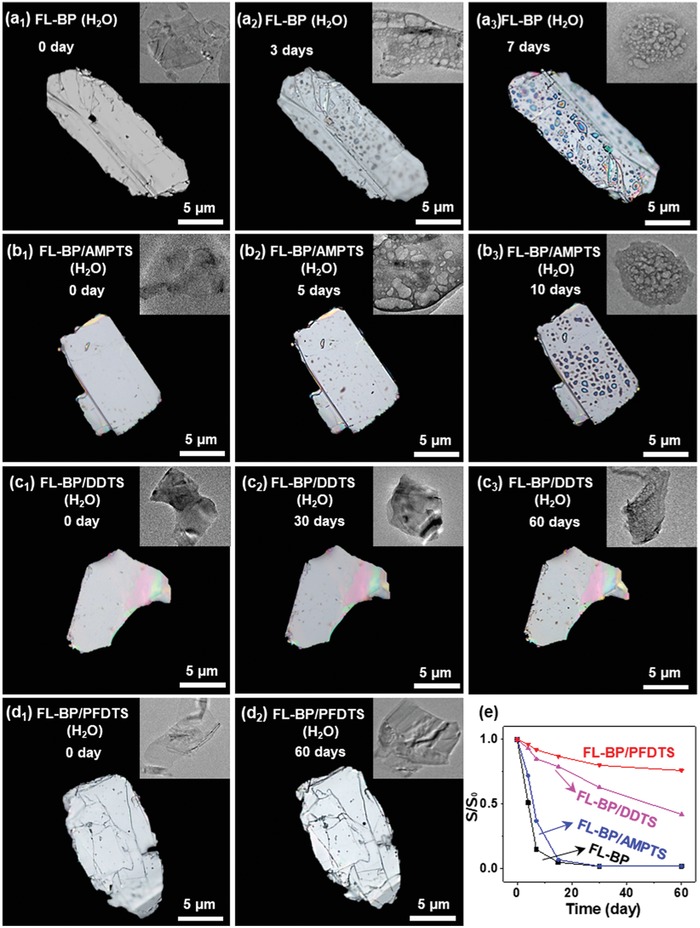
Polarizing microscope images of a) FL‐BP, b) FL‐BP/AMPTS, c) FL‐BP/DDTS, and d) FL‐BP/PFDTS exposed in high moisture content environment for different duration as indicated (Inset: the corresponding TEM image). e) Conductivity variation of FL‐BP, FL‐BP/AMPTS, FL‐BP/DDTS, and FL‐BP/PFDTS exposed in high moisture content environment for different duration.

Then, the stability of as‐prepared FL‐BP (0.98 ± 0.08 µm in thickness, Figure S13a, Supporting Information), FL‐BP/AMPTS (1.00 ± 0.07 µm in thickness, Figure S13b, Supporting Information), FL‐BP/DDTS (1.02 ± 0.08 µm in thickness, Figure S13c, Supporting Information) and FL‐BP/PFDTS (1.01 ± 0.09 µm in thickness, Figure S13d, Supporting Information) are compared by polarizing microscope and TEM characterization (**Figure** 4). FL‐BP, FL‐BP/AMPTS, FL‐BP/DDTS, and FL‐BP/PFDTS were dropped on the glass slide, and then kept in high moisture content environment at room temperature for different durations. In the initial stage, the optical images of all fresh prepared four samples show perfectly clean and flat surfaces (Figure 4a_1_,b_1_,c_1_,d_1_). TEM images of these four samples present the same 2D FL‐BP nanosheet structures without observed defects (inset of Figure 4a_1_,b_1_,c_1_,d_1_). The surface of FL‐BP and FL‐BP/AMPTS becomes rough with small topographic protrusions (hereafter termed “bubbles”) due to the formation of P*_x_*O*_y_* after 3 and 5 days, respectively.^37^ Size of the bubbles increases after 7 days and surface becomes rougher after 10 days. The corresponding TEM images of FL‐BP and FL‐BP/AMPTS show bubbles obviously on the surface after 3 and 5 days (inset of Figure 4a_2_,b_2_). 2D FL‐BP and FL‐BP/AMPTS nanostructures are completely destroyed and only bubbles are observed after 7 and 10 days respectively (inset of Figure 4a_3_,b_3_). In contrary to fast degradation of FL‐BP and FL‐BP/AMPTS, the surface of FL‐BP/DDTS only becomes lightly rough after 60 days (Figure 4c_3_). The surface morphology of FL‐BP/PFDTS is preserved and no obvious bubbles, corrosion, or degradation can be observed after 60 days (Figure 4d_2_) under the same conditions. Even after high moisture content environment exposure for 90 days, the FL‐BP/PFDTS does not show significant degradation, which is confirmed by polarizing microscope (Figure S14a, Supporting Information), TEM (Figure S14b, Supporting Information), and Raman spectra (Figure S14c, Supporting Information). The results show the great improvement on the stability of BP via DDTS (hydrophobic) coating and PFDTS (amphiphobic) coating in high moisture content environment.

The conductivity of four samples was further addressed to evaluate their stability. The conductivity of FL‐BP and FL‐BP/AMPTS falls rapidly (10% and 27% remaining) upon high moisture content environment exposure for 7 days, whereas the conductivity of FL‐BP/DDTS and FL‐BP/PFDTS remain around 51% and 74% after 2 months (Figure 4e). The retained conductivity of FL‐BP/PFDTS also indicates the enhanced stability arisen from amphiphobic coating.

**Figure 5:**
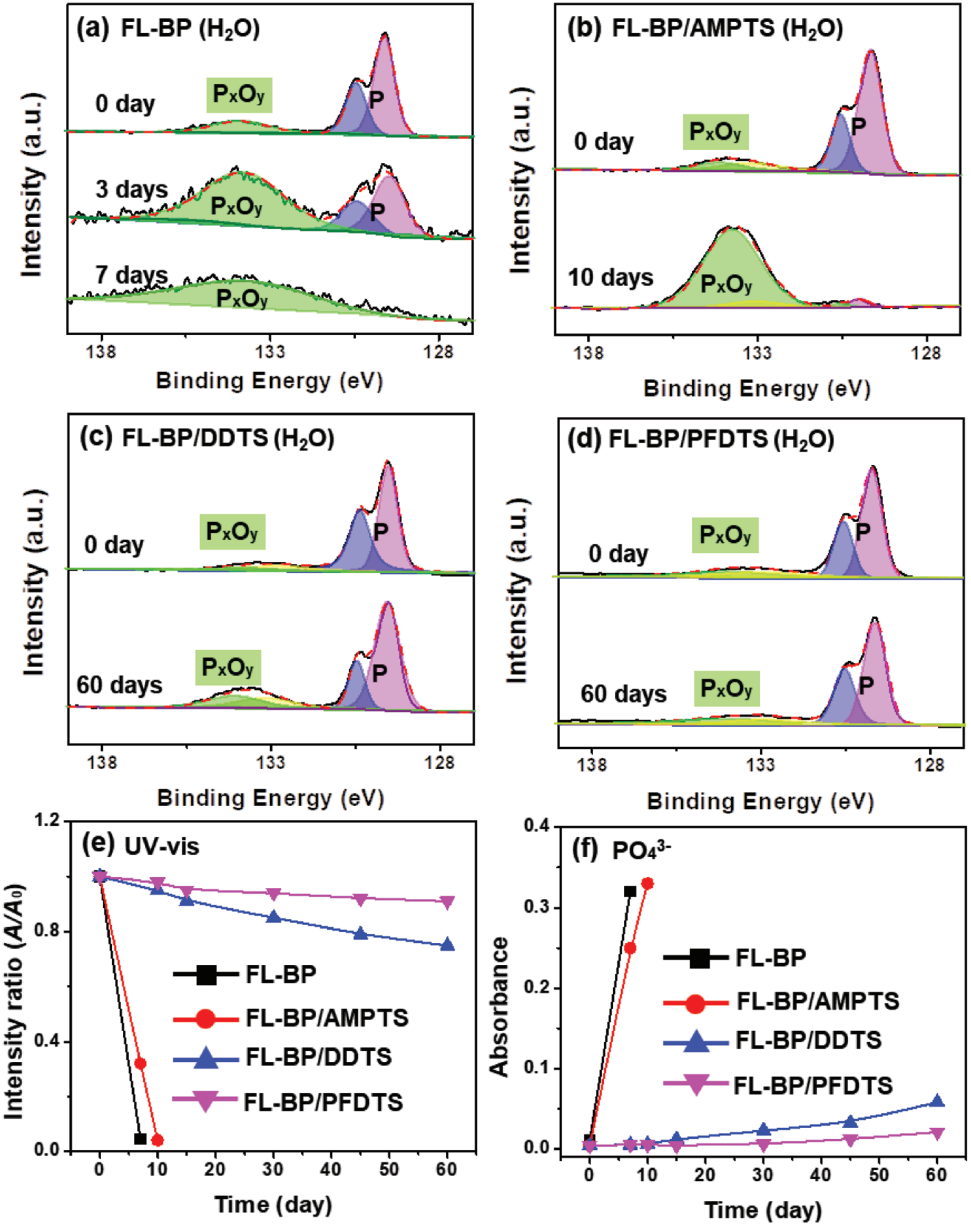
HR‐XPS spectra of P 2p peaks for a) FL‐BP, b) FL‐BP/AMPTS, c) FL‐BP/DDTS, and d) FL‐BP/PFDTS in high moisture content environment for different durations. e) Variation of the absorption ratios at 470 nm (*A/A*_0_) of FL‐BP, FL‐BP/AMPTS, FL‐BP/DDTS, and FL‐BP/PFDTS incubated in water for different durations (*A*_0_: original value). f) UV–vis adsorption variation of PO_4_^3−^ at 710 nm, detected in FL‐BP, FL‐BP/AMPTS, FL‐BP/DDTS, and FL‐BP/PFDTS aqueous solution for different durations. For (e) and (f), see original UV–vis spectra in Figures S16 and S17 in the Supporting Information.

The degradation of FL‐BP initiates from the reaction between P and oxygen, subsequently forms oxidized phosphorus species (FL‐BP → P*_x_*O*_y_*), and finally converts into P*_x_*O*_y_* (FL‐BP → P*_x_*O*_y_* → PO_4_^3−^).[qv: 8c] Hence, the contents of P*_x_*O*_y_* and PO_4_^3−^ are supposed to be increasing along with the degradation time of FL‐BP. Since both FL‐BP/DDTS and FL‐BP/PFDTS are demonstrated to be more stable than FL‐BP and FL‐BP/AMPTS, the content of P*_x_*O*_y_* and PO_4_^3−^ on the surface of FL‐BP/DDTS and FL‐BP/PFDTS is supposed to be less than that of FL‐BP and FL‐BP/AMPTS. To confirm this, XPS characterization is utilized to address the variation of P*_x_*O*_y_* and PO_4_^3−^ content during the degradation of four samples in high moisture content environment. The P 2p XPS spectra for FL‐BP, FL‐BP/AMPTS, FL‐BP/DDTS, and FL‐BP/PFDTS are shown in **Figure** 5a–d, respectively. In the high‐resolution P 2p XPS spectrum of each sample, two peaks at 129.6 and 130.5 eV are assigned to elemental phosphorus, and the other peak at 134.1 eV is assigned to P*_x_*O*_y_*.[qv: 9a] For the as‐prepared FL‐BP, the peak intensity of elemental phosphorus drops significantly (P: from 82.52% to 39.76%) for 3 days (Figure 5a), and finally becomes very weak (P: 2.31%) after 7 days (Figure 5a). Meanwhile, for the same sample (the as‐prepared FL‐BP), the peak intensity of P*_x_*O*_y_* becomes stronger (P*_x_*O*_y_*: from 17.48% to 60.24%) after 3 days, and remains at high level (P*_x_*O*_y_*: 97.69%) after 7 days, indicating the heavy oxidation of FL‐BP. Similarly, for FL‐BP/AMPTS, the peak intensity of elemental phosphorus becomes very weak (P: from 83.54% to 3.60%) and the peak intensity of P*_x_*O*_y_* becomes stronger (P*_x_*O*_y_*: from 9.15% to 90.09%) after 7 days (Figure 5b). In sharp contrast, for FL‐BP/DDTS and FL‐BP/PFDTS, the peak intensity of elemental phosphorus is strong, while the peak of P*_x_*O*_y_* is weak (Figure 5c,d). These two peaks have no significant changes even after 60 days (as shown in Table S2 in the Supporting Information). Such results illustrate that the DDTS and PFDTS functionalization gives a great improvement on the stability of FL‐BP in high moisture content environment.

To further prove the improved stability of FL‐BP/DDTS and FL‐BP/PFDTS, UV–vis spectra are conducted to monitor the variation of FL‐BP concentration, as well as corresponding PO_4_^3−^ concentration (Figure S15 in the Supporting Information for more details^37^) during the degradation of FL‐BP in aqueous solution. At initial stage, the same concentration (7 µg mL^−1^) of four samples dispersion gives the almost same UV–vis absorbance intensity at 470 nm. With dispersing time increases, the absorbance intensity at 470 nm (*A*) decreases by 96.0% (as compared with the original value *A*_0_) for FL‐BP after 7 days, and 95.6% for FL‐BP/AMPTS after 10 days (Figure 5e). The absorbance intensity of PO_4_^3−^ increases from 0.01 to 0.26 after 7 days for FL‐BP and from 0.01 to 0.24 after 10 days for FL‐BP/AMPTS (Figure 5f). The result suggests the fast degradation of FL‐BP and FL‐BP/AMPTS in aqueous solution. On the contrary, absorbance intensity at 470 nm of FL‐BP/DDTS slightly decreases (25.0%) and the concentration of PO_4_^3−^ increases a bit (0.01 to 0.058) after 60 days. For FL‐BP/PFDTS, the absorbance intensity of FL‐BP (9.0%), as well as the concentration of PO_4_^3−^ (0.01 to 0.021) remain at the same level, even after 60 days. Obviously, both XPS and UV–vis demonstrate the great stability improvement of FL‐BP via DDTS or PFDTS coating, which is consistent with the polarizing microscope and TEM results as previously discussed.

**Figure 6:**
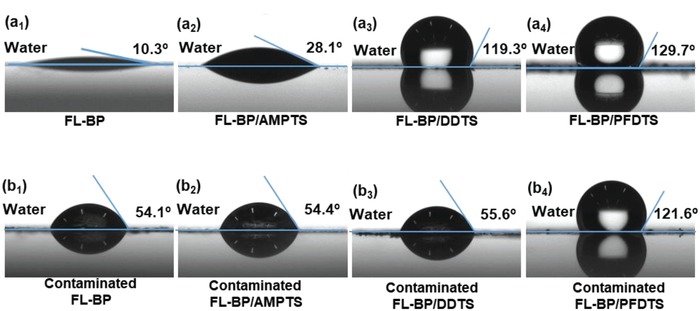
a) Water contact angles of FL‐BP, FL‐BP/AMPTS, FL‐BP/DDTS, and FL‐BP/PFDTS. b) Water contact angles of the contaminated FL‐BP, the contaminated FL‐BP/AMPTS, the contaminated FL‐BP/DDTS, and the contaminated FL‐BP/PFDTS (oleic acid as the contaminant).

Based on the above results, both hydrophobic surface and amphiphobic surface render FL‐BP with high stability in high moisture content environment. However, hydrophobic surfaces normally lose their water repellency when contaminated by small amount of oil or organic solvent.[qv: 12a,b] As previously reported, water is one of the key factors that determine the degradation of FL‐BP.[qv: 8c] Once the coating layers lose its water repellency, the efficiency of passivation would be low. To mimic the contamination, a thin layer of oleic acid was cast on the surface of FL‐BP and three types of functionalized FL‐BP. The surface property changes are disclosed by measuring the water contact angles of contaminated FL‐BP, contaminated FL‐BP/AMPTS, contaminated FL‐BP/DDTS, and contaminated FL‐BP/PFDTS, respectively (**Figure** 6). The water contact angles of the contaminated FL‐BP and the contaminated FL‐BP/AMPTS are 54.1° and 54.4°, respectively (Figure 6b_1_,b_2_), which are more hydrophobic than the initial FL‐BP (10.3°) and the initial FL‐BP/AMPTS (28.1°). The water contact angle of the contaminated FL‐BP/DDTS decreases significantly from 119.3° to 55.6°. The reason for such change of the contaminated FL‐BP/DDTS is attributed to the low surface energy of oil. The surface tension of the oil is lower than that of water, resulting in the oil penetrating through the surfaces to decrease the interfacial tension sufficiently.[qv: 12b,d,38] The water contact angle of the contaminated FL‐BP/PFDTS is almost the same as that of the original FL‐BP/PFDTS, indicating that the hydrophobic surface of FL‐BP/PFDTS retains due to its oleophobic property.

**Figure 7:**
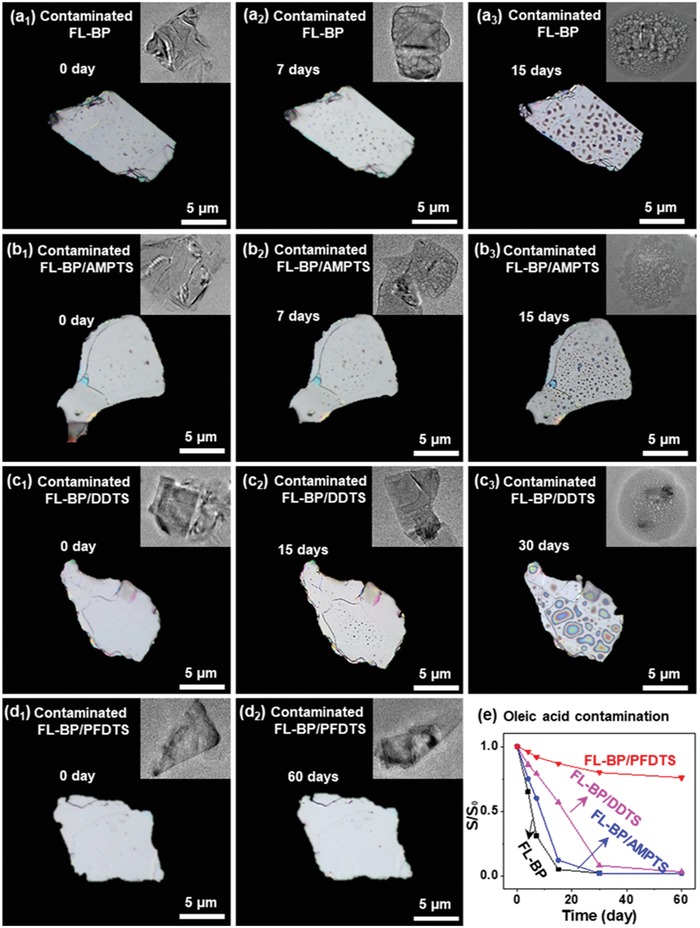
Stability study after the surface contamination by oleic acid. Polarizing microscope images of a) the contaminated FL‐BP, b) the contaminated FL‐BP/AMPTS, c) the contaminated FL‐BP/DDTS, and d) the contaminated FL‐BP/PFDTS exposed in high moisture content environment for different durations as indicated (Inset: corresponding TEM image). e) Conductivity variation of the contaminated FL‐BP, the contaminated FL‐BP/AMPTS, the contaminated FL‐BP/DDTS, and the contaminated FL‐BP/PFDTS exposed in aqueous environment for different durations.

The stability of the contaminated FL‐BP, the contaminated FL‐BP/AMPTS, the contaminated FL‐BP/DDTS, and the contaminated FL‐BP/PFDTS in high moisture content environment is further investigated by polarizing microscope and TEM (**Figure** 7). At initial stage, all contaminated four samples have perfectly clean and flat surfaces (Figure 7a_1_,b_1_,c_1_,d_1_). TEM images of these contaminated four samples show the same 2D FL‐BP nanosheet structures without observed defects (inset of Figure 7a_1_,b_1_,c_1_,d_1_). The surfaces of the contaminated FL‐BP, the contaminated FL‐BP/AMPTS, and the contaminated FL‐BP/DDTS become rough and generate a mass of bubbles after incubation for 7, 7, and 15 days, respectively (Figure 7a_2_,b_2_,c_2_). TEM images (inset of Figure 7a_2_,b_2_,c_2_) further confirm bubbles on the surface of the corresponding samples. The surfaces of the contaminated FL‐BP, the contaminated FL‐BP/AMPTS, and the contaminated FL‐BP/DDTS show abundant bubbles (Figure 7a_3_,b_3_,c_3_) after 15, 15, and 30 days, respectively. The 2D structures of these three types of FL‐BP are almost destroyed (inset of Figure 7a_3_,b_3_,c_3_). For the contaminated FL‐BP/PFDTS, the morphology shows no significant changes during incubation. Even after 60 days, the 2D nanostructure remains perfectly as indicated by TEM (Figure 7d). Such results demonstrate that PFDTS functionalization renders FL‐BP with enhanced stability in high moisture content environment although the surface of nanosheets is contaminated by oleic acid. The conductivity of the contaminated FL‐BP/PFDTS only experiences a slight decrease (75% remaining) in high moisture content environment even after 2 months (Figure 7e), while the conductivity of other contaminated samples drops rapidly under the same conditions. Such retained conductivity also suggests the enhanced stability of amphiphobic FL‐BP under the contamination.

**Figure 8:**
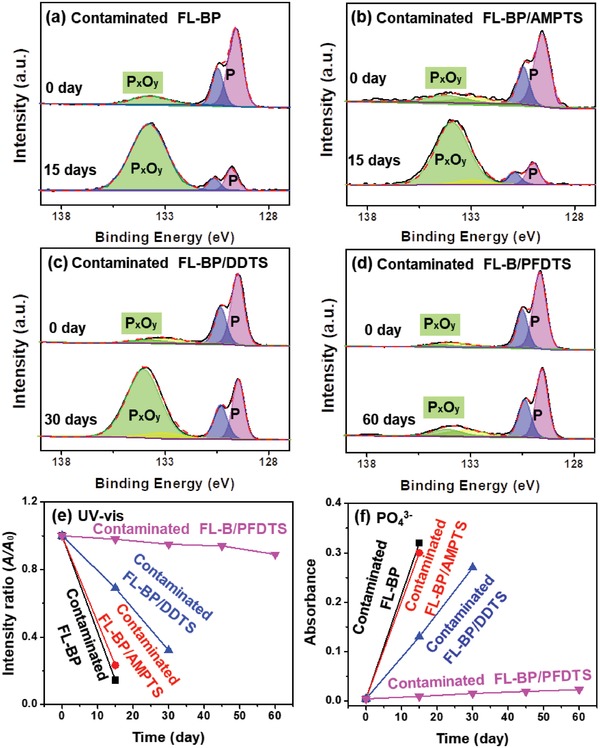
Stability study after the surface contamination by oleic acid. HR‐XPS spectra of P 2p peaks for a) the contaminated FL‐BP, b) the contaminated FL‐BP/AMPTS, c) the contaminated FL‐BP/DDTS, and d) the contaminated FL‐BP/PFDTS in high moisture content environment for different durations. e) Variation of the UV–vis absorption ratios at 470 nm (*A/A*_0_) for the contaminated FL‐BP, the contaminated FL‐BP/AMPTS, the contaminated FL‐BP/DDTS, and the contaminated FL‐BP/PFDTS incubated in water for different durations (*A*_0_: original value). f) UV–vis adsorption variation of PO_4_^3−^ at 710 nm, detected in the contaminated FL‐BP, the contaminated FL‐BP/AMPTS, the contaminated FL‐BP/DDTS, and the contaminated FL‐BP/PFDTS dispersion incubated in water for different durations. For (e) and (f), see original UV–vis spectra in Figures S18 and S19 in the Supporting Information.

XPS spectra are also utilized for addressing the degradation of four contaminated samples in high moisture content environment. For the contaminated FL‐BP, the contaminated FL‐BP/AMPTS, and the contaminated FL‐BP/DDTS, the peak intensity of elemental phosphorus drops significantly after 15, 15, and 30 days, respectively (**Figure** 8a–c). Meanwhile, the peak intensity of P*_x_*O*_y_* for all three samples becomes stronger after 15, 15, and 30 days, indicating significant oxidation of the contaminated FL‐BP (P: from 83.03% to 14.53%; P*_x_*O*_y_*: from 16.97% to 85.47%), the contaminated FL‐BP/AMPTS (P: from 77.78% to 12.40%; P*_x_*O*_y_*: from 12.77% to 78.13%), and the contaminated FL‐BP/DDTS (P: from 85.78% to 31.44%; P*_x_*O*_y_*: from 7.65% to 62.50%). In contrast, for the contaminated FL‐BP/PFDTS at initial stage, the peak intensity of elemental phosphorus is strong, while the peak of P*_x_*O*_y_* is weak (Figure 8d). These two peaks have no significant change even after 60 days (see details in Table S3 in the Supporting Information). XPS analysis indicates the stability of contaminated FL‐BP/PFDTS under high moisture content environment is significantly higher than that of the other three types of FL‐BP nanosheets.

The stability of the contaminated FL‐BP/PFDTS in aqueous solution is also studied by UV–vis spectra (Figure 8e,f). At initial stage, the same concentration (7 µg mL^−1^) of four contaminated samples gives almost the same UV–vis absorbance intensity of BP at 470 nm (Figure 8e). Upon time, the absorbance intensity at 470 nm (*A*) decreases by 85.7% (as compared with the original value *A*_0_) for the contaminated FL‐BP after 15 days, 76.8% for the contaminated FL‐BP/AMPTS after 15 days, and 68.0% for the contaminated FL‐BP/DDTS after 30 days, respectively (Figure 8e). The absorbance intensity of PO_4_^3−^ increases from 0.01 to 0.26 after 15 days for the contaminated FL‐BP, from 0.01 to 0.25 after 15 days for the contaminated FL‐BP/AMPTS, and from 0.01 to 0.23 after 30 days for the contaminated FL‐BP/DDTS (Figure 8f). In strong contrast, for the contaminated FL‐BP/PFDTS, the absorbance intensity of FL‐BP nanosheets, as well as the concentration of PO_4_^3−^ remains at the same level, even after 60 days (Figure 8e,f). It is noted that the stability of the contaminated FL‐BP (15 days) and the contaminated FL‐BP/AMPTS (15 days) is superior to the initial FL‐BP (7 days) and the initial FL‐BP/AMPTS (7 days), probably because that oleic acid or organic solvents can form solvation shell to protect FL‐BP.[qv: 11a] Such results demonstrate that, upon contaminated by a small amount of oleic acid, FL‐BP/DDTS becomes significantly less stable in high moisture content environment or aqueous solution, while FL‐BP/PFDTS still maintains high stability. UV–vis spectral analysis is consistent with the observations from polarizing microscope, TEM, and XPS results. Similar phenomenon can be observed when the surfaces of FL‐BP and the three types of functionalized FL‐BP are contaminated by other organic reagents (such as CH_2_Cl_2_ and *N*‐methyl‐2‐pyrrolidone, more details in Figure S20 in the Supporting Information).

## 3 Conclusion

In conclusion, the robust amphiphobic FL‐BP is achieved by surface coating of fluorinated surfactant PFDTS in colloidal solution. Attributed to the amphiphobic surface, the obtained FL‐BP/PFDTS demonstrates strong surface water‐repellency, even its surface being contaminated by oil liquid or other organic solvents (such as oleic acid, CH_2_Cl_2_ and *N*‐methyl‐2‐pyrrolidone). The morphology, the crystal structure, as well the conductivity of the FL‐BP/PFDTS demonstrate no significant difference from the originally as‐prepared FL‐BP, even if exposed under harsh conditions (including high moisture content environment in the presence/absence of oil or other organic solvents) for 2 months. Amphiphobic functionalization provides an effective strategy for passivation of FL‐BP, from which the future practical applications can benefit.

## 4 Experimental Section

Experimental details are presented in the Supporting Information.

## Conflict of Interest

The authors declare no conflict of interest.

## Supporting information

Supplementary
