# Supplementary material for: Robust Amphiphobic Few‐Layer Black Phosphorus Nanosheet with Improved Stability
Source: Adv Sci (Weinh). 2019 Sep 30;6(23):1901991. doi: 10.1002/advs.201901991 (PMC6891918; doi:10.1002/advs.201901991)
Supplement: Supplementary file 1 — Supplementary [file ADVS-6-1901991-s001.pdf]

Copyright WILEY-VCH Verlag GmbH & Co. KGaA, 69469 Weinheim, Germany, 2019.

ADVANCED SCIENCE
Open Access

Supporting Information

for *Adv. Sci.,* DOI: 10.1002/advs.201901991

Robust Amphiphobic Few-Layer Black Phosphorus Nanosheet with Improved Stability

Xiao Liu, Yunfei Bai, Jun Xu, Qingchi Xu, Liangping Xiao, Liping Sun, Jian Weng, and Yanli Zhao**

Supporting Information

Experimental Section

Materials: (3-Aminopropyl)trimethoxysilane (AMPTS), red phosphorus (RP), tin, and tin iodide (SnI_4) were purchased from Aladdin (Shanghai, China). 1H,1H,2H,2H-Perfluorooctyltrichlorosilane (PFDTS) and trichloro(dodecyl)silane (DDTS) were obtained from Alfa (Shanghai, China). Other chemicals such as N,N-dimethylformamide (DMF) and ethanol were purchased from Xilong Chemical Co., Ltd. (Shantou, China). All chemicals were used without further purification. Organic solvents are degassed and distilled prior to use for stability experiments.

Preparation of bulk BP: Bulk BP was prepared according to the literature.[11] In a typical synthesis, red phosphorus (500 mg), Sn (20 mg), and SnI_4 (10 mg) were sealed in a quartz tube. The tube was heated to 923 K with a heating ramp rate of about 1.35 K per min and held at 923 K for five hours. The tube was then ramped down to 773 K with rate of 0.33 K per min, followed by a natural cooling process. The large BP crystals were collected and washed with hot toluene and acetone for several times to remove the residual mineralizer. After being dried under vacuum, the product was kept in a glovebox for further analysis.

Preparation of FL-BP/AMPTS, FL-BP/DDTS and FL-BP/PFDTS: FL-BP was prepared using a simple liquid exfoliation technique. In brief, bulk BP (20 mg) was dispersed in DMF (40 mL) that bubbled with argon to eliminate the dissolved oxygen molecules for oxidation prevention. The solution was sonicated with ice bath for 24 h. Then, the brown suspension was centrifuged at 2000 rpm for 20 min to remove the unexfoliated BP residual and the supernatant was collected for further usage. Before use, the FL-BP was spun down at 12000 rpm for 20 min to remove DMF. The obtained FL-BP was added to the AMPTS (5 mmol/L) ethanol solution, DDTS (5 mmol/L) ethanol solution, PFDTS (1 mmol/L) DMF solution for 24 h, respectively. After the completion of reaction, the precipitated product was separated from the supernatant by centrifugation, which was washed with ethanol for several times. The precipitated FL-BP/AMPTS, FL-BP/DDTS, and FL-BP/PFDTS was collected for use in subsequent experiments.

Preparation of contaminated FL-BP, contaminated FL-BP/AMPTS, contaminated FL-BP/DDTS and contaminated FL-BP/PFDTS: the obtained FL-BP, FL-BP/AMPTS, FL-BP/DDTS, and FL-BP/PFDTS precipitates were dried, and immersed in oleic acid for 24 h. Finally, contaminated FL-BP, contaminated FL-BP/AMPTS, contaminated FL-BP/DDTS and contaminated FL-BP/PFDTS were collected by centrifugation, and directly used in subsequent experiments.

Stability measurements: The stability of samples was investigated via polarizing microscope, XPS, TEM, UV-vis, and Raman. In high moisture content environment (95% humidity),

large-sized FL-BP (form sediment after sonicated with ice bath for 24 h) was dropped on the glass slide, then the change of surface morphology was observed via polarizing microscope intuitively for different duration. Micro-sized FL-BP (8000 rpm) was dropped on the silicon substrate, then peak of P and the other peak of P_xO_y was observed via XPS for different duration. In aqueous solution, the stability of micro-sized FL-BP (8000 rpm) was investigated via TEM, UV-vis, and Raman for different duration.

Conductivity measurements: Conductivity measurements of FL-BP (566 ± 72 nm in size, and 2.64 ± 0.26 nm in thickness) and FL-BP/PFDTS (574 ± 48 nm in size, and 3.71 ± 0.27 nm in thickness) are conducted as following: The aqueous solution of samples was dropped onto the glass substrates, and dried in vacuum at 60 °C for 1 h. Then, the current-voltage curves were generated via cyclic voltammetry using a quartz crystal microbalance (CHI440C: Initial E (V) = -1, High E (V) = 1, Low E (V) = -1, Scan Rate (V/s) = 0.1, Sensitivity (A/V) = $1e^{-5}$).

Zeta potential measurements: The samples were dispersed in an aqueous solution with sonication for 30 min at 300 W. Then Zeta potential was measured by Malvern Nano−ZS.

Characterization of samples: The obtained powder was characterized by Fourier transform infrared spectrometer (NICOLET iS10). The micrographs of samples were taken by transmission electron microscope (TEM, JEOL JEM-2100), high resolution TEM (HRTEM, TECNAI F-30), transmission-reflecting polarizing microscope (ECLIPSE/Ci-S, Nikon), scanning electron microscopy (SEM, SU70, Hitachi). AFM images were recorded using an atomic force microscope (AFM, DI Multimode V/DI Multimode V, Veeco) in tapping mode. X-ray photoelectron spectroscopy (XPS) measurements were performed on a PHI Quantum 2000 (USA). X-ray diffraction (XRD, Philips X'Pert Pro, Philips, Amsterdam, Netherlands; λ = 1.54056 Å) was performed with Cu Ka radiation. The optical properties of FL-BP nanosheets were characterized by UV-visible spectrophotometer (UV-2550, Shimadzu). Thermal gravimetric analysis (TG) was performed on an SDT-Q600 instrument under nitrogen atmosphere at a heating rate of 10 °C min^{-1}. Raman spectra (XploRA, Jobin-Yvon) were recorded with a solid-state laser at the excitation wavelength of 532 nm. The current-voltage curves were generated by a quartz crystal microbalance (CHI440C).

Supplementary Figures

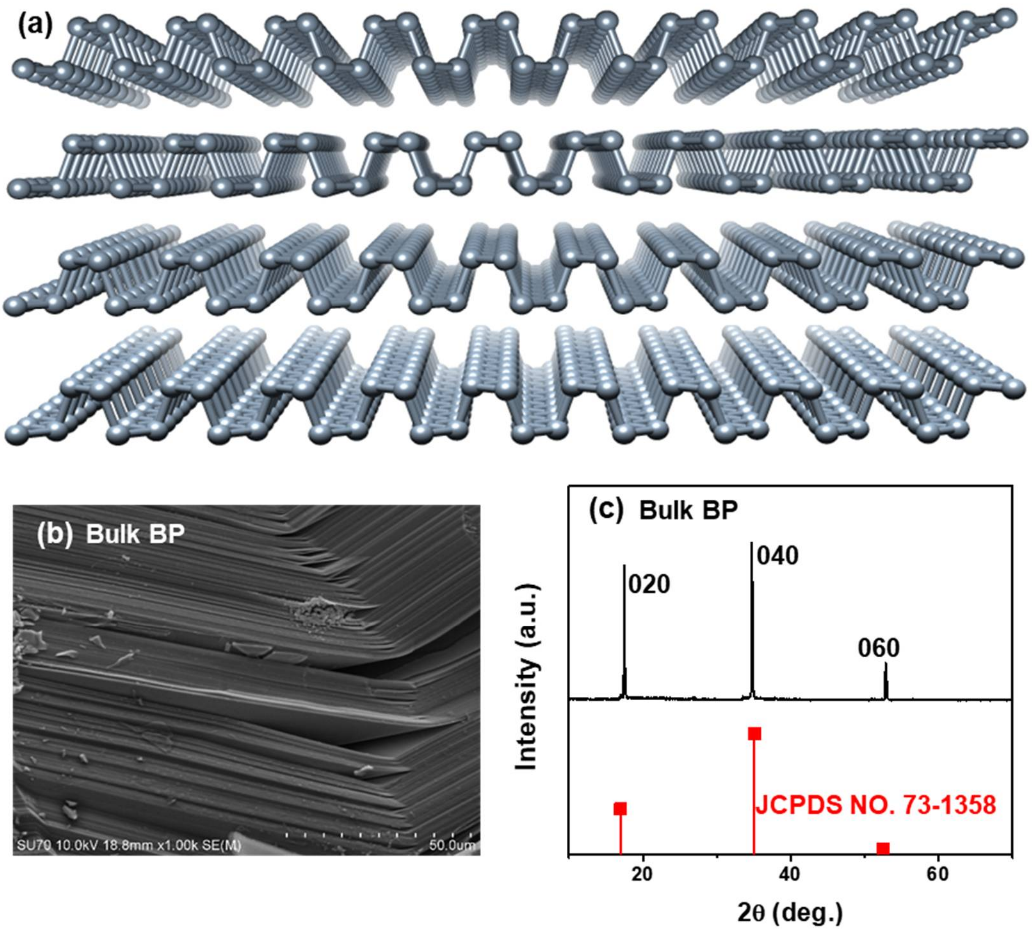

Figure S1. a) Atomic structure of BP. b) SEM image of the as-prepared bulk BP showing the layered structure of BP. c) XRD patterns of the as-prepared bulk BP. Bar diagram for the JCPDS of BP.

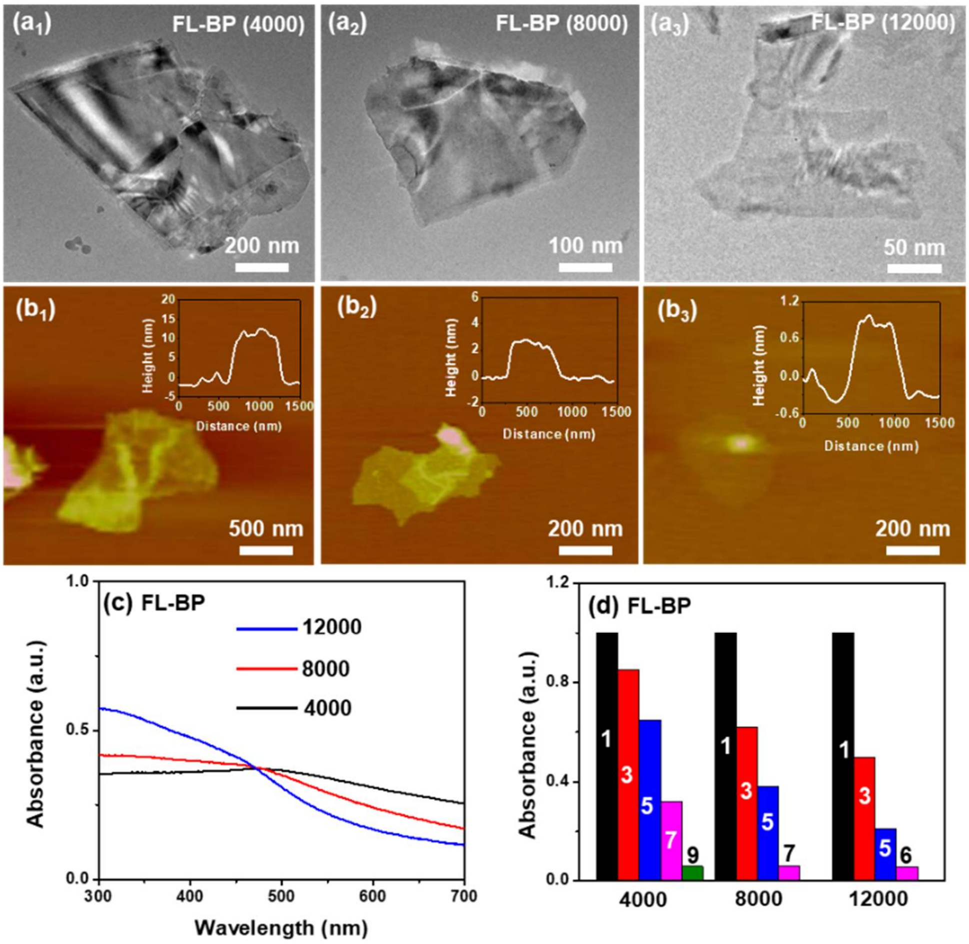

Figure S2. The as-exfoliated FL-BP isolated with different centrifugal speeds. a) TEM and b) AFM images, Inset in Figure Sb_1-b_3: the corresponding height profiles of FL-BP. c) UV-vis absorption spectra of FL-BP in DMF. The absorption region generated blue-shifted with decreasing thickness and size of FL-BP. d) The stability of FL-BP decreases with thickness and size of FL-BP.

Preparation details: Firstly, the stock solution was centrifuged at 2000 rpm for 20 min, and the precipitate was removed. The remaining supernatant was centrifuged at 4000 rpm for 20 min, and the precipitate was collected, which named as FL-BP-4000. Then, the supernatant collected from FL-BP-4000 was further centrifuged at 8000 rpm for 20 min, and the precipitate was collected, which named as FL-BP-8000. Lastly, the supernatant collected from FL-BP-8000 was further centrifuged at 12000 rpm for 20 min, and the precipitate was collected, which named as FL-BP-12000.

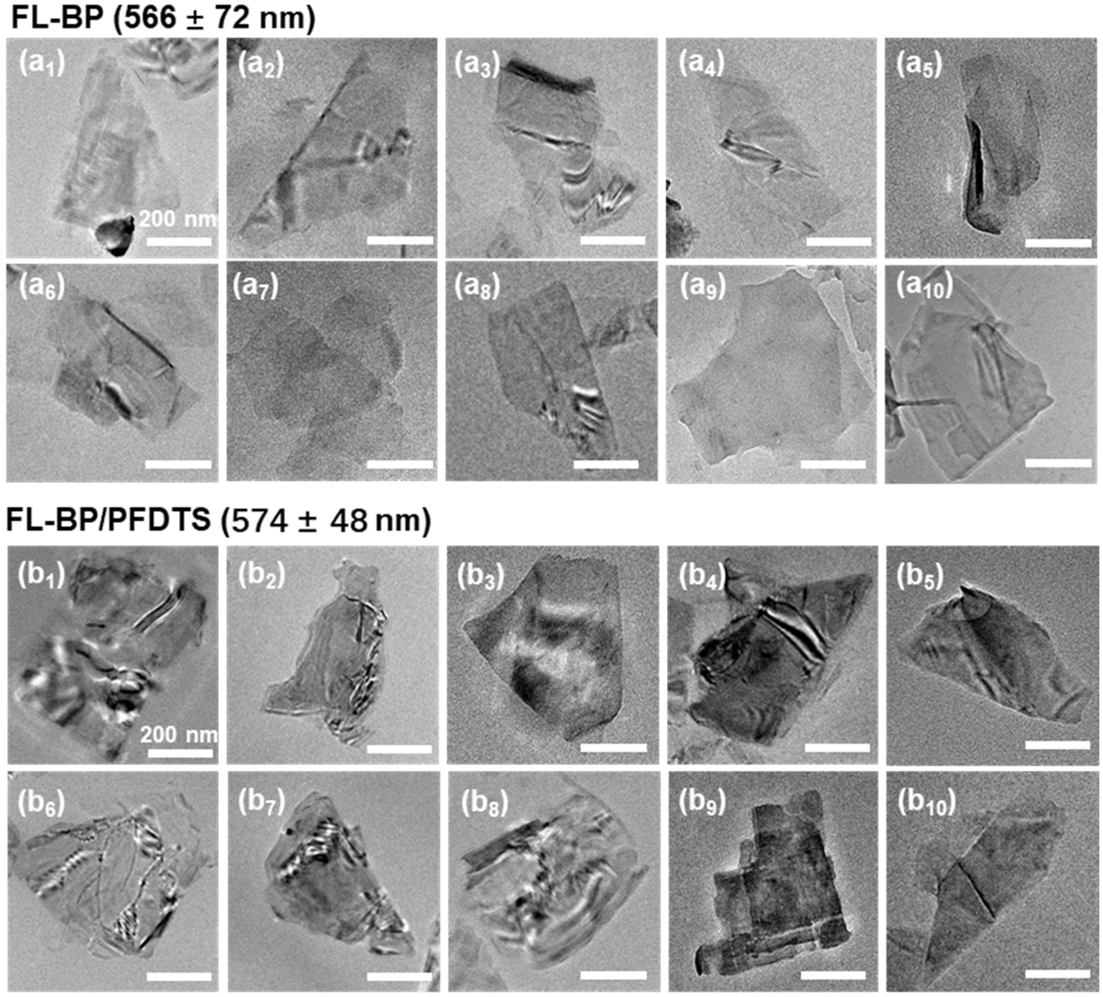

Figure S3. Statistical TEM images of a) FL-BP and b) FL-BP/PFDTS.

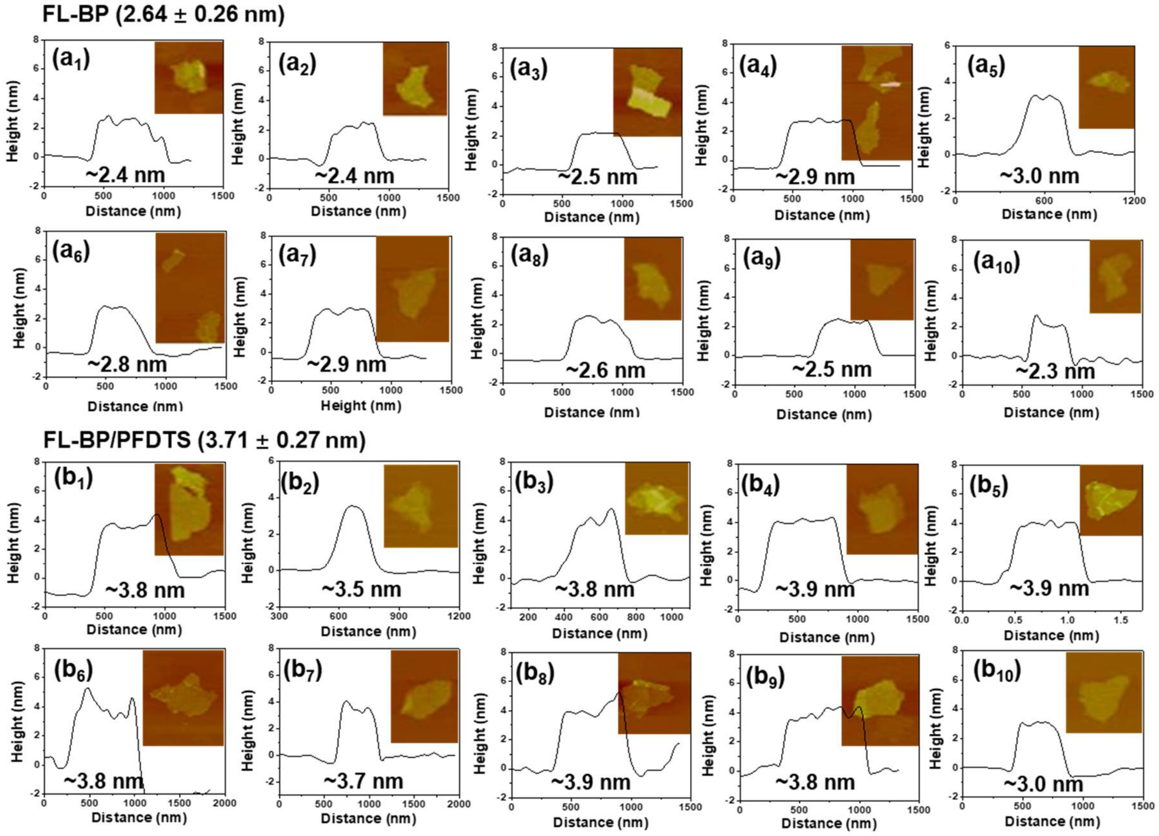

Figure S4. Statistical AFM images of a) FL-BP and b) FL-BP/PFDTS.

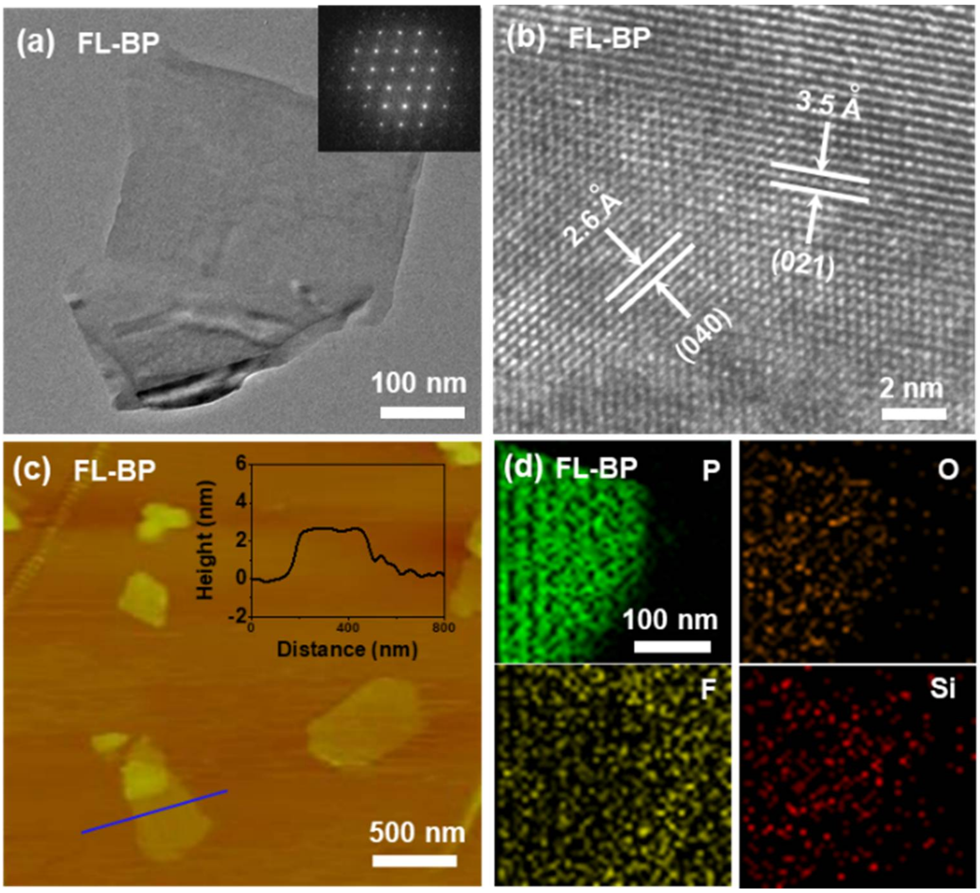

Figure S5. a) TEM image of FL-BP (Inset: SAED pattern), b) HRTEM image of FL-BP and c) AFM image of FL-BP with height profile insertion. d) HAADF-STEM and elemental mapping of FL-BP.

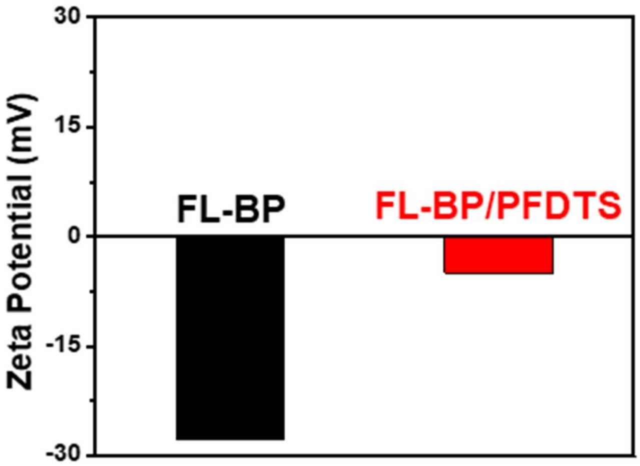

Figure S6. Zeta potential of FL-BP and FL-BP/PFDTS.

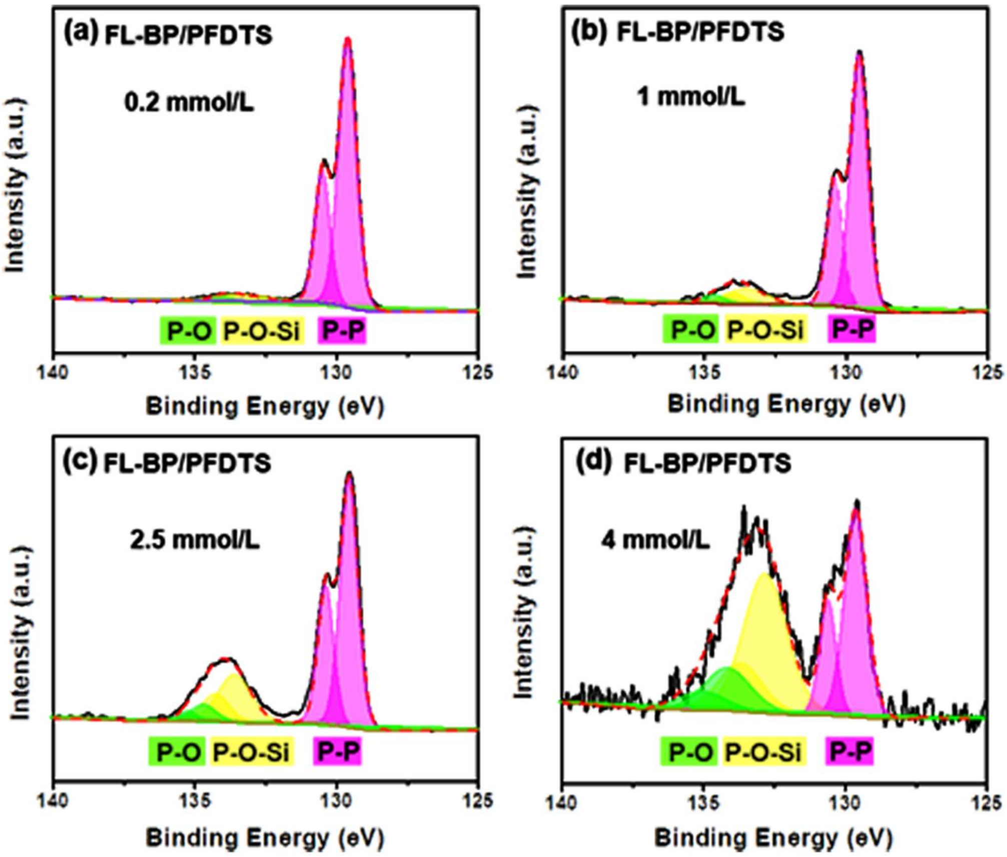

Figure S7. High resolution XPS spectra for P 2p peaks of FL-BP/PFDTS with different concentrations of PFDTS: a) 0.2 mmol/L, b) 1 mmol/L, c) 2.5 mmol/L, and d) 4 mmol/L.

Table S1. Summary of the composition of different bonds in the samples shown in Figure S7.

Sample	P-P (%)	P-O-Si (%)	P-O (%)
FL-BP/PFDTS (0.2 mmol/L)	94.67	4.00	1.33
FL-BP/PFDTS (1 mmol/L)	86.39	11.24	3.37
FL-BP/PFDTS (2.5 mmol/L)	70.56	23.84	5.60
FL-BP/PFDTS (4 mmol/L)	36.81	47.23	15.96

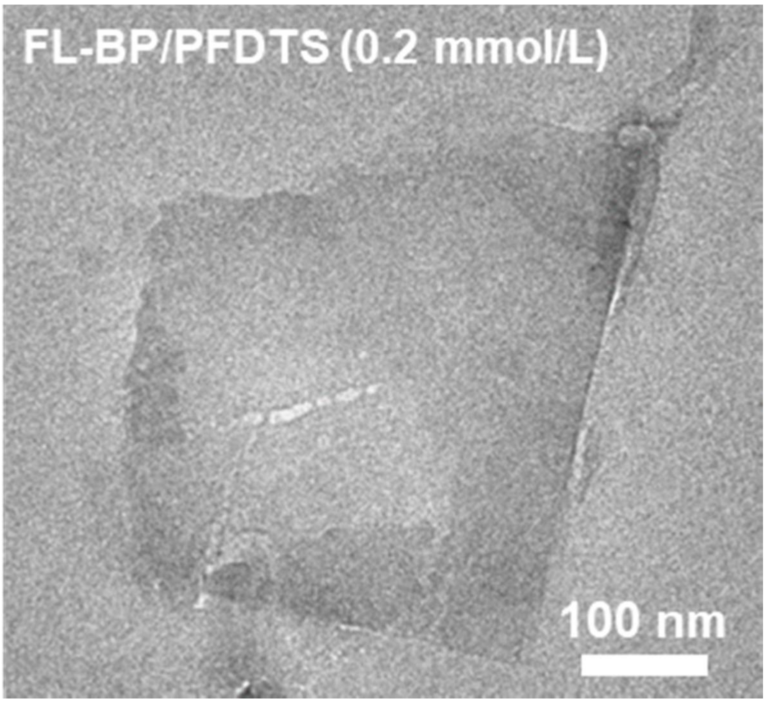

Figure S8. TEM image of FL-BP/PFDTS (PFDTS 0.2 mmol/L)

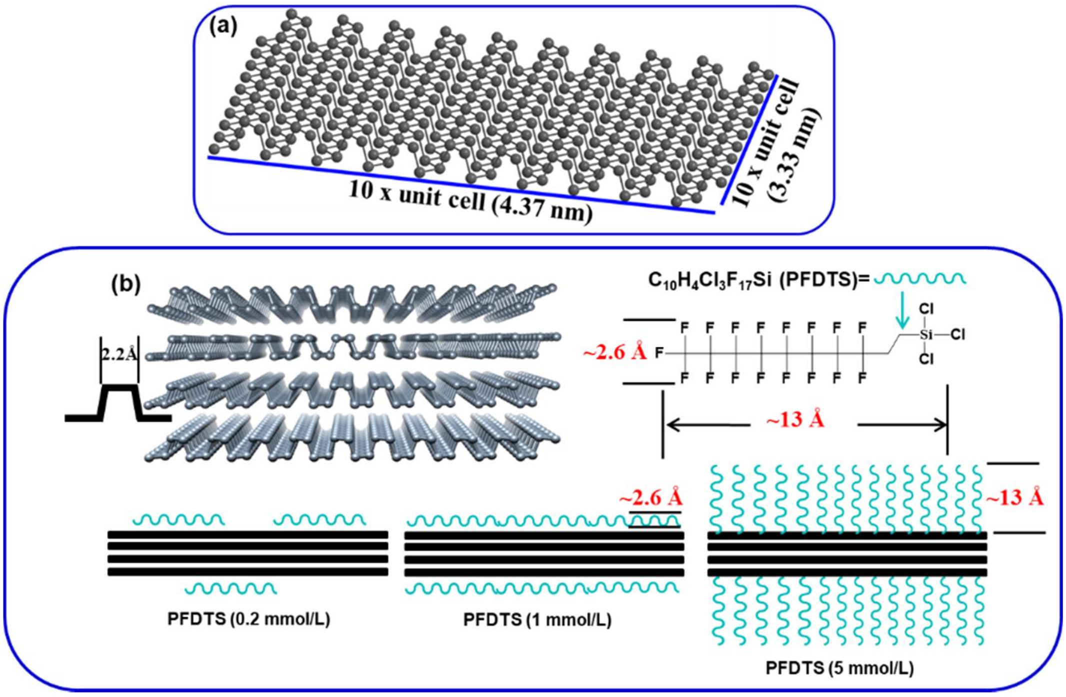

Figure S9. a) Model of BP unit cell and monolayer BP sheet (10 unit cell × 10 unit cell). b) Theoretical calculation model.

In Figure S9, there are 400 phosphorus atoms taken into account in a monolayer BP sheet (10 unit cell × 10 unit cell), whose area is 14.55 nm^2. It can be calculated that there are 274900 phosphorus atoms in a monolayer BP sheet with area of 100 × 100 nm^2. It is taken a four-layered BP with 100 nm side length for each layer as a calculation model. As illustrated in Figure S9, almost 10 phosphorus atoms correspond to one PFDTS molecule when thin PFDTS layer on the surface of FL-BP and almost 4 phosphorus atoms correspond to one PFDTS molecule when thick PFDTS layer on the surface of FL-BP. (N_A= Avogadro's constant).

Mass of phosphorus: $274900 \times 4 \times 31/N_A \approx 34 \times 10^6/N_A$
(thin layer) Mass of $C_{10}H_4F_{17}SiO_2$: $274900/12 \times 2 \times 490/N_A \approx 22 \times 10^6/N_A$
(thin layer) Mass of SiO_2: $274900/12 \times 2 \times 60/N_A \approx 2.7 \times 10^6/N_A$
(thick layer) Mass of $C_{10}H_4F_{17}SiO_2$: $274900/6 \times 2 \times 490/N_A \approx 45 \times 10^6/N_A$
(thick layer) SiO_2: $274900/6 \times 2 \times 60/N_A \approx 5.5 \times 10^6/N_A$

Hence, FL-BP/PFDTS (PFDTS 1 mmol/L) contains ~4.8% SiO_2 and FL-BP/PFDTS (PFDTS 5 mmol/L) contains ~6.9% SiO_2 by theoretical calculation.
(thin layer) Mass of F: $274900/12 \times 2 \times 17 \times 19/N_A \approx 14 \times 10^6/N_A$
Atomic ratio of P/F=1.36:1

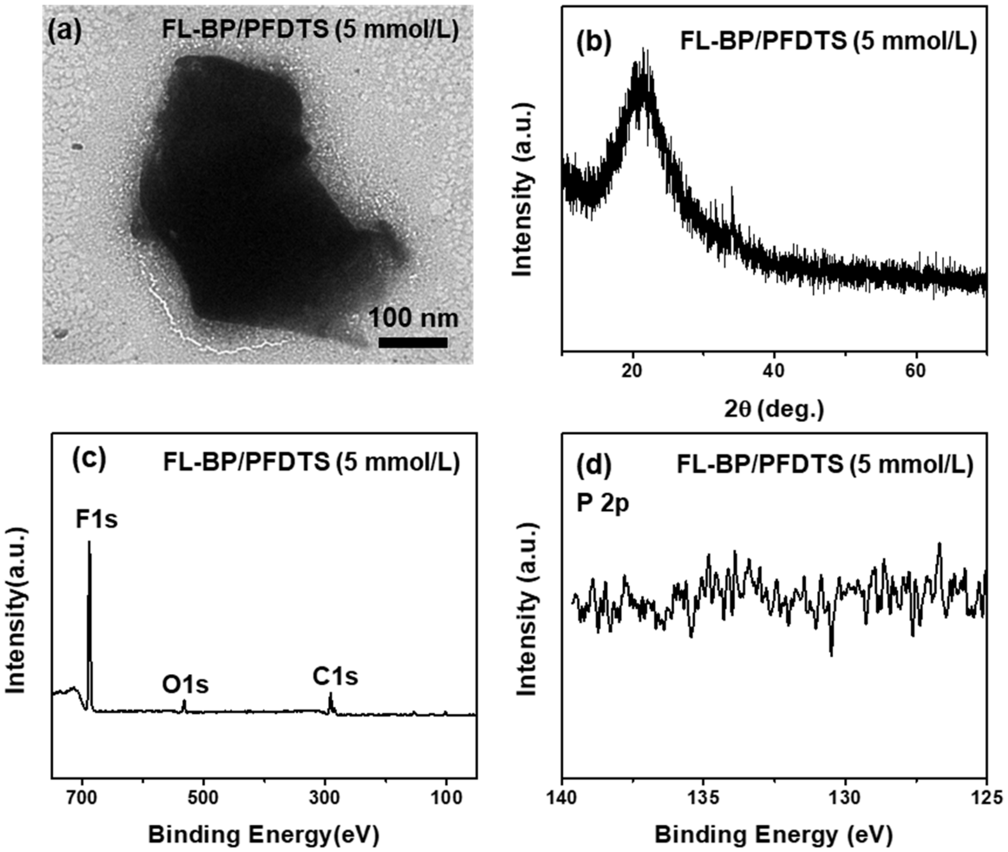

Figure S10. a) TEM image, b) XRD and c) XPS of FL-BP/PFDTS (PFDTS 5 mmol/L). d) High resolution XPS spectra for P 2p peaks of FL-BP/PFDTS (PFDTS 5 mmol/L).

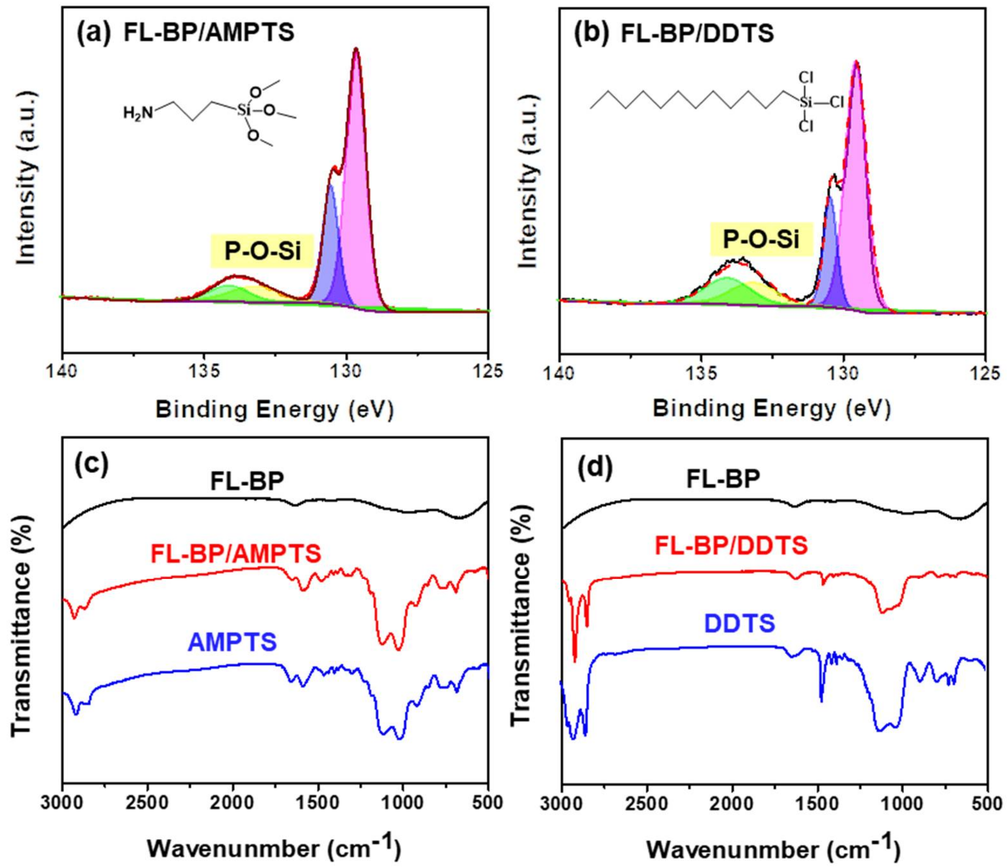

Figure S11. High resolution XPS spectra of P 2p peaks of a) FL-BP/AMPTS and b) FL-BP/DDTS. c) FTIR spectra of FL-BP, FL-BP/AMPTS, and AMPTS. d) FTIR spectra of FL-BP, FL-BP/DDTS, and DDTS.

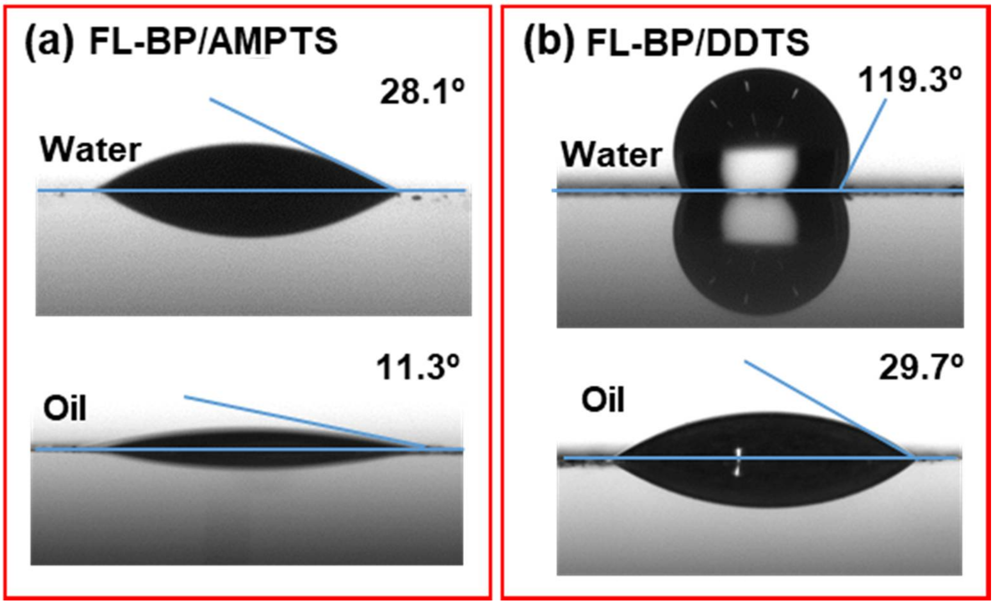

Figure S12. a) Water contact angle and oil contact angle of FL-BP/AMPTS. b) Water contact angle and oil contact angle of FL-BP/DDTS.

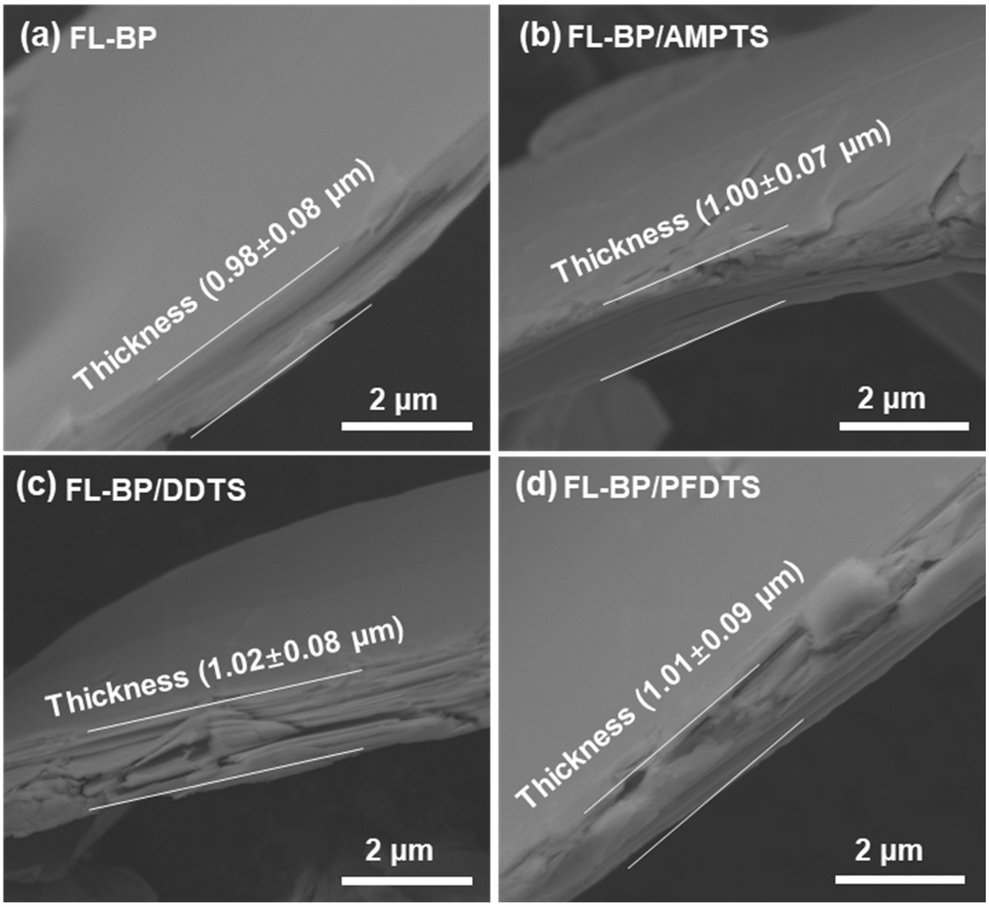

Figure S13. SEM images of a) FL-BP (0.98 ± 0.08 μm in thickness), b) FL-BP/AMPTS (1.00 ± 0.07 μm in thickness), c) FL-BP/DDTS (1.02 ± 0.08 μm in thickness), and d) FL-BP/PFDTS (1.01 ± 0.09 μm in thickness).

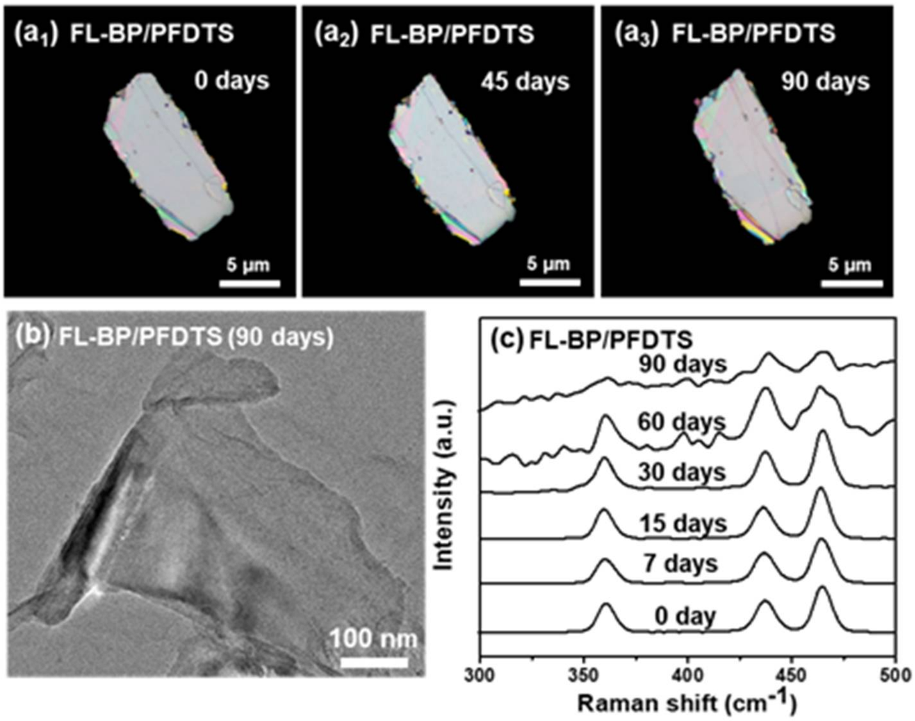

Figure S14. a) Polarizing microscope images of FL-BP/PFDTS after exposure in high moisture content environment. b) TEM images of FL-BP/PFDTS after incubation for 90 days in aqueous solution. c) Raman spectra of FL-BP/PFDTS in aqueous solution for different duration.

Table S2. Summary of the composition of different P in the samples shown in Figure 5.

Sample	P (%)			P_xO_y (%)		
FL-BP	0 day	3 days	7 days	0 day	3 days	7 days
	82.52	39.76	2.31	17.48	60.24	97.69
FL-BP/AMPTS	0 day	10 days		0 day	10 days	
	83.54	3.6		9.15	90.09	
FL-BP/DDTS	0 day	60 days		0 day	60 days	
	83.43	76.19		5.12	11.24	
FL-BP/PFDTS	0 day	60 days		0 day	60 days	
	84.15	83.61		4.72	6.21	

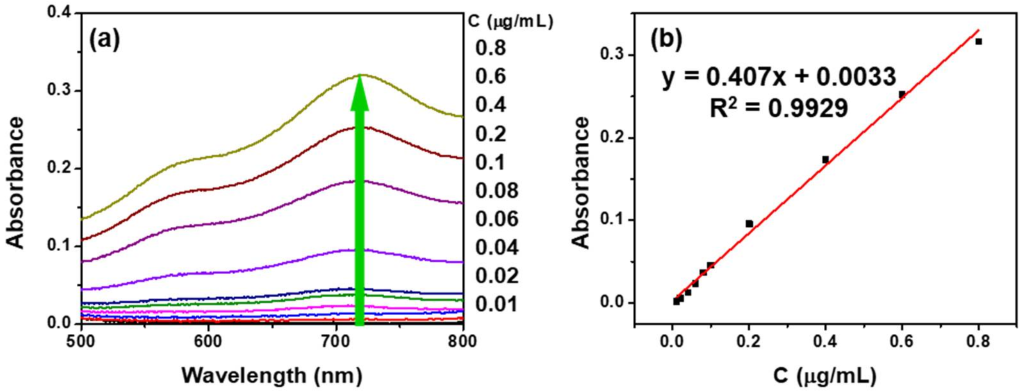

Figure S15. a) UV-vis absorption spectra of the mixed solution with different concentrations of PO_4^{3-}. b) Plot of absorbance as a function of concentration.

Solution A - H_2SO_4 solution (H_2SO_4:H_2O = 1:1)
Solution B - $K_2S_2O_8$ solution (50 g/L)
Solution C - Vitamin C solution (100 g/L)
Solution D - Molybdate solution

Preparation of molybdate solution: 100 mL $(NH_4)_6Mo_7O_{24}\cdot4(H_2O)$ solution (0.13 g/mL) and 100 mL $C_8H_4K_2O_{12}Sb_2$ solution (0.0035g/mL) are slowly added to the 300 mL Solvent A.

Solution E - K_2HPO_4 solution (2 μg/mL).

In this paper, PO_4^{3-} was detected via Ammonium molybdate spectrophotometric method (*China Water Wastewater* **2006**, *22*, 85-86). PO_4^{3-} reacts with $NH_4^+MoO_4^{2-}$ to form $(MoO_2\cdot4MoO_3)_2H_3PO_4$ under acidic condition (Vitamin C as reducing agent). The absorption peak is at 710 nm, which is a characteristic absorption of $(MoO_2\cdot4MoO_3)_2H_3PO_4$.

$$PO_4^{3-} + NH_4^+ MoO_4^{2-} + H^+ \rightarrow (NH_4)_3PO_4\cdot12MoO_3+H_2O$$

$$(NH_4)_3PO_4\cdot12MoO_3+ \text{Vitamin C solution} + H^+ \rightarrow (MoO_2\cdot4MoO_3)_2H_3PO_4$$

Standard curve (absorption intensity at 710 nm v.s. PO_4^{3-} concentration): The linear detection range for PO_4^{3-} spans a concentration range of 0.01~0.6 μg/mL. 0.01, 0.02, 0.04, 0.06, 0.08, 0.1, 0.2, 0.4, 0.6 and 0.8 μg/mL E solutions (K_2HPO_4 10 mL) were prepared. 2 mL B solution ($K_2S_2O_8$) was added to each E solution (K_2HPO_4) for phosphite oxidation to phosphate and then each E solution (K_2HPO_4) was heated at 120 °C for 30 min in an autoclave, respectively. After cooling down to room temperature, 1 mL C solution (Vitamin C) and 2 mL D solution (Molybdate) were subsequently added to each E solution (K_2HPO_4). After 15 min, the UV-vis of mixed solution was measured and the absorbance of 710 nm corresponds to amount of PO_4^{3-} (as shown Figure S15).

Amount of PO_4^{3-} from FL-BP and FL-BP/PFDTS aqueous solution was detected via ammonium molybdate spectrophotometric method. The FL-BP (7 μg/mL) and FL-BP/PFDTS (7 μg/mL) dispersed in water for different periods of time were centrifuged at 15000 rpm for 20 min to collect the supernatant, respectively. Each supernatant (1 mL) collected was diluted to ten folds to meet the detection range of 0.01~0.6 μg/mL. Then, the amount of PO_4^{3-} of supernatants was determined by the standard curve.

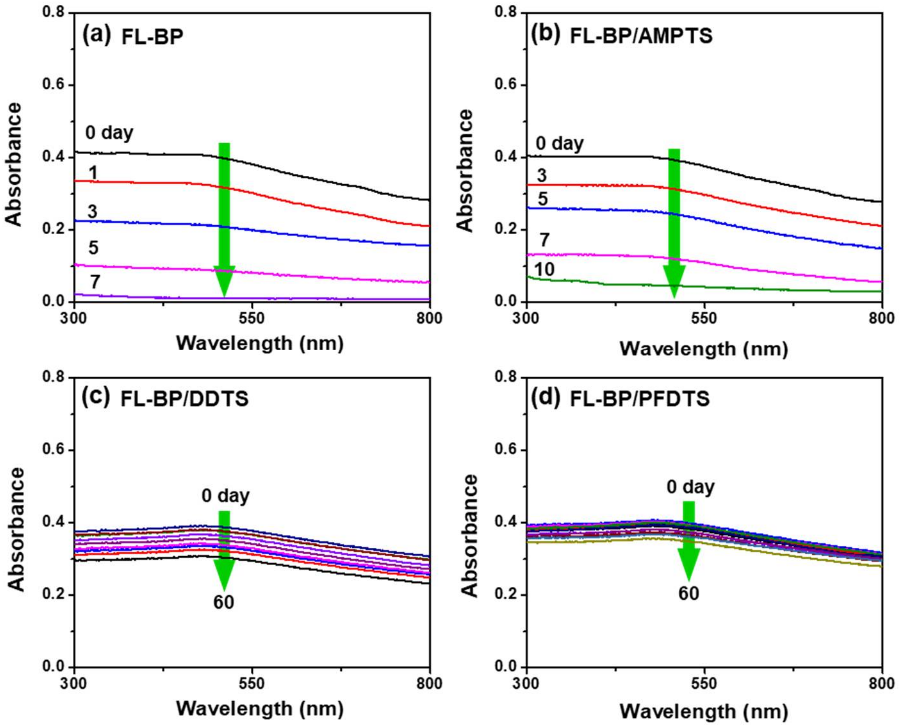

Figure S16. a-d) UV-vis spectra of FL-BP, FL-BP/AMPTS, FL-BP/DDTS, and FL-BP/PFDTS in aqueous solution for different duration.

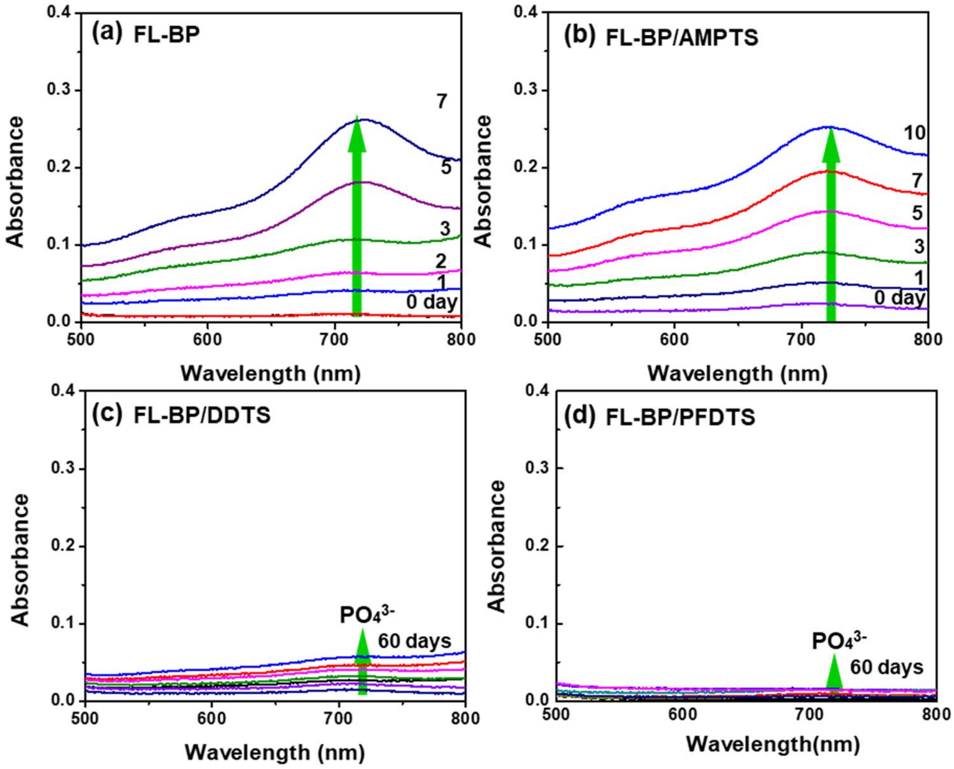

Figure S17. a-d) UV-vis spectra of PO_4^{3-} in FL-BP, FL-BP/AMPTS, FL-BP/DDTS, and FL-BP/PFDTS aqueous solution for different duration.

Table S3. Summary of the composition of different P in the samples shown in Figure 8.

Sample	P (%)		P_xO_y (%)	
Contaminated FL-BP	0 day	15 days	0 day	15 days
	83.03	14.53	16.97	85.47
Contaminated FL-BP/AMPTS	0 day	15 days	0 day	10 days
	77.78	12.40	12.77	78.13
Contaminated FL-BP/DDTS	0 day	60 days	0 day	60 days
	85.79	31.44	7.65	62.50
Contaminated FL-BP/PFDTS	0 day	60 days	0 day	60 days
	86.75	75.36	3.13	11.11

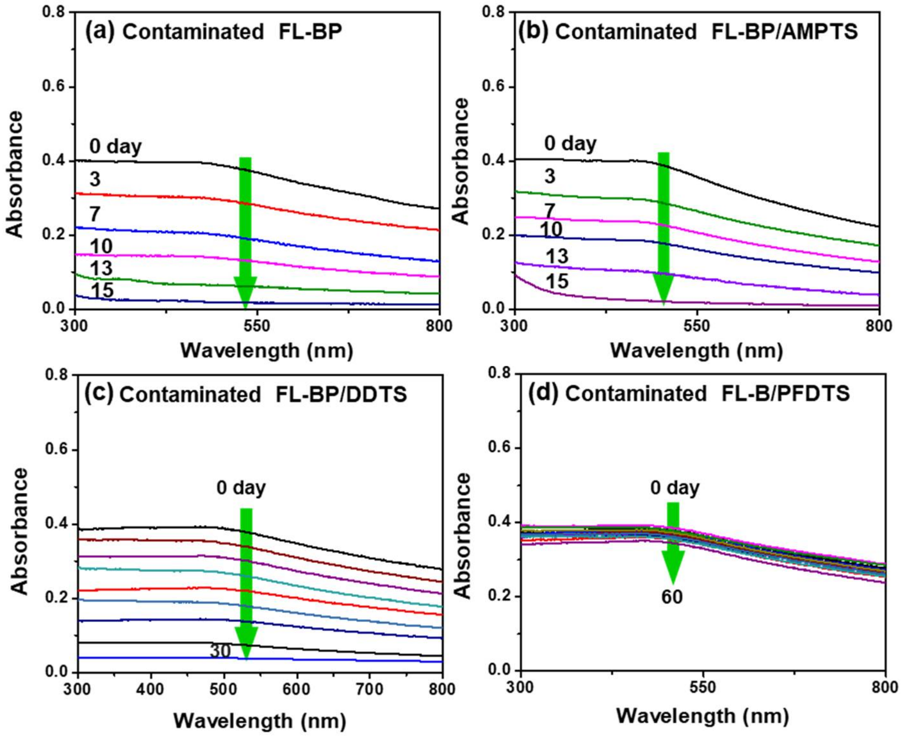

Figure S18. a-d) UV-vis spectra of the contaminated FL-BP, the contaminated FL-BP/AMPTS, the contaminated FL-BP/DDTS, and the contaminated FL-BP/PFDTS in aqueous solution for different duration.

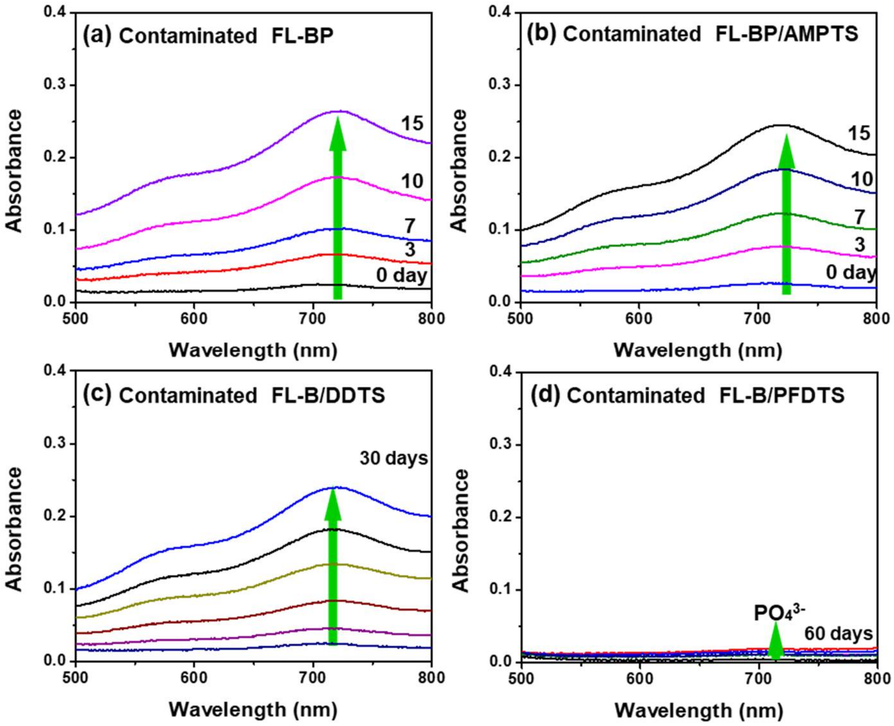

Figure S19. a-d) UV-vis spectra of PO_4^{3-} in the contaminated FL-BP, the contaminated FL-BP/AMPTS, the contaminated FL-BP/DDTS, and the contaminated FL-BP/PFDTS aqueous solution for different duration.

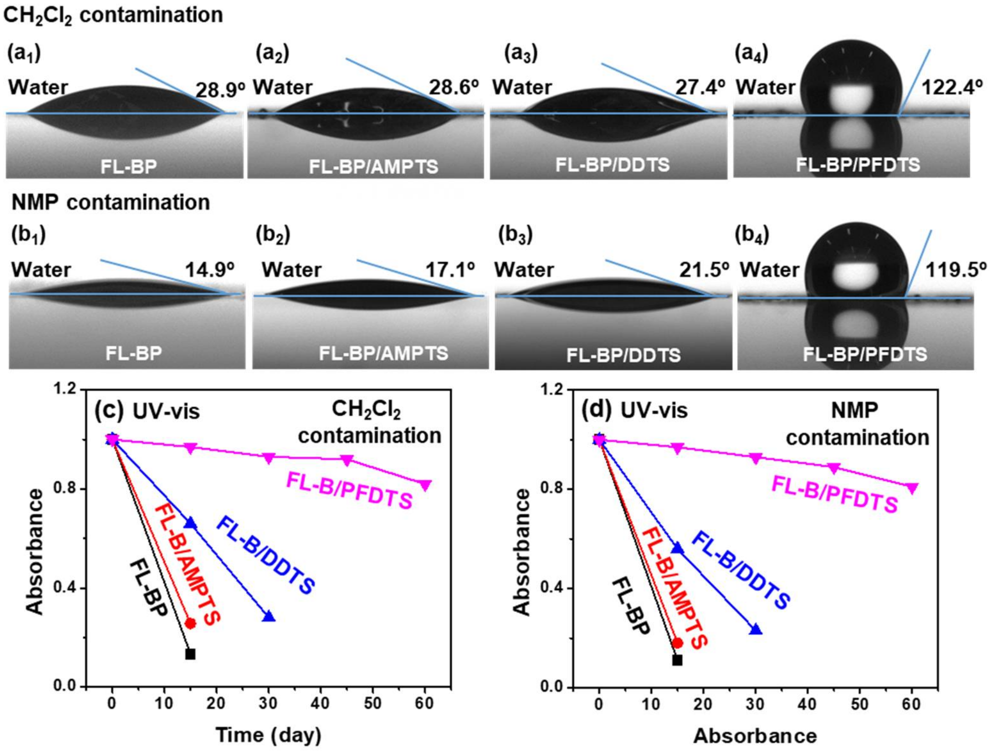

Figure S20. a) Water contact angle of FL-BP, FL-BP/AMPTS, FL-BP/DDTS, and FL-BP/PFDTS after CH_2Cl_2 contamination. b) Water contact angle of FL-BP, FL-BP/AMPTS, FL-BP/DDTS, and FL-BP/PFDTS after *N*-methyl-2-pyrrolidone contamination. Variation of UV-Vis adsorption at 470 nm of FL-BP, FL-BP/AMPTS, FL-BP/DDTS, and FL-BP/PFDTS after c) CH_2Cl_2, and d) *N*-methyl-2-pyrrolidone contamination.
